# Molecular Dynamics Simulations with Grand-Canonical Reweighting Suggest Cooperativity Effects in RNA Structure Probing Experiments

**DOI:** 10.1021/acs.jctc.3c00084

**Published:** 2023-06-08

**Authors:** Nicola Calonaci, Mattia Bernetti, Alisha Jones, Michael Sattler, Giovanni Bussi

**Affiliations:** † Scuola Internazionale Superiore di Studi Avanzati, SISSA, via Bonomea 265, Trieste 34136, Italy; ‡ Department of Mathematics and Geosciences, University of Trieste, Trieste 34127, Italy; ¶ Institute of Structural Biology, Helmoltz Zentrum Muünchen, Neuherberg 85764, Germany; § Bavarian NMR Center at Department of Chemistry, Technical University of Munich, Garching 85757, Germany; ∥ Center for Integrated Protein Science München and Bavarian NMR Center at Department of Chemistry, Technical University of Munich, Garching 85757, Germany

## Abstract

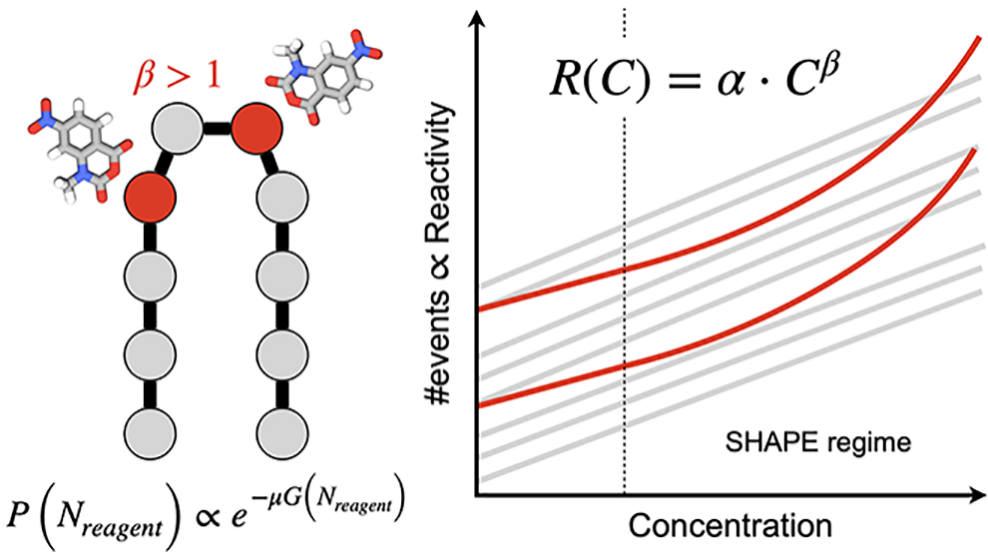

Chemical probing experiments such as SHAPE are routinely used to probe RNA molecules. In this work, we use atomistic molecular dynamics simulations to test the hypothesis that binding of RNA with SHAPE reagents is affected by cooperative effects leading to an observed reactivity that is dependent on the reagent concentration. We develop a general technique that enables the calculation of the affinity for arbitrary molecules as a function of their concentration in the grand-canonical ensemble. Our simulations of an RNA structural motif suggest that, at the concentration typically used in SHAPE experiments, cooperative binding would lead to a measurable concentration-dependent reactivity. We also provide a qualitative validation of this statement by analyzing a new set of experiments collected at different reagent concentrations.

## 1 Introduction

**Figure 1:**
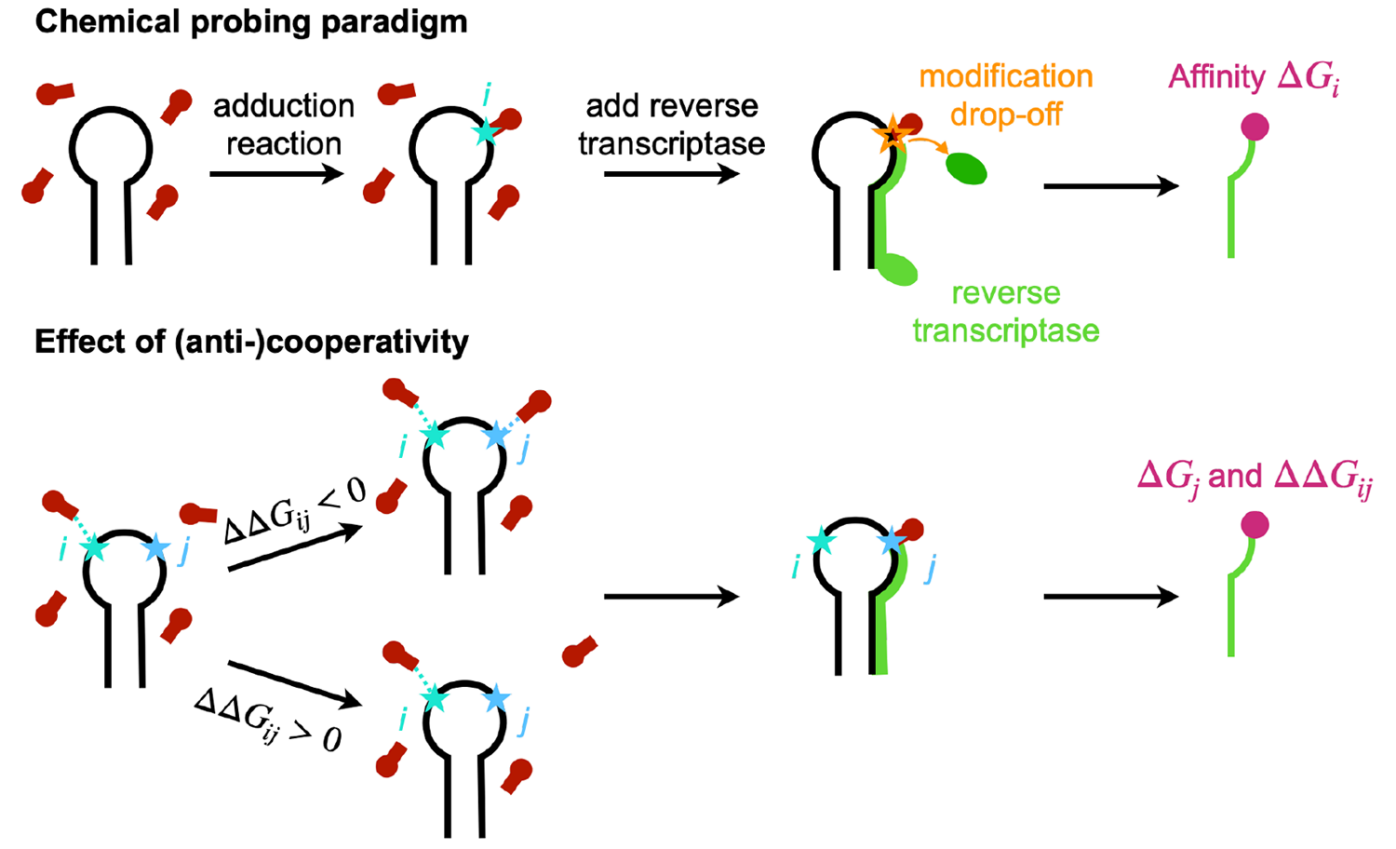
Chemical probing paradigm and effects of cooperativity. In chemical probing experiments (upper panel), RNA is treated with a reagent that binds covalently. Binding is assumed to be related to a structural determinant that depends on the specific reagent. Reverse transcription or other techniques are then used to detect which nucleotides were reactive and thus infer structural properties of the probed motif. Cooperativity or anticooperativity effects might impact observed reactivities (lower panel). In particular, when experiments are performed at a finite reagent concentration, a nonlinear dependence of reactivity on reagent concentration is possible. We notice that chemical binding is not required for this effect to be visible. Even in a single hit kinetics approximation, where a single adduction per RNA molecule is observed, multiple reagent copies might physically interact with each other and with RNA, acting as small molecular crowders perturbing its structural dynamics.

Chemical probing experiments allow measuring RNA structure at nucleotide resolution by adding a chemical reagent to RNA in solution and probing at which positions adducts are formed.^1^ A prototypical case is the selective 2′-hydroxyl acylation analyzed by the primer extension (SHAPE) technique,^2^ where reagents bind to the hydroxyl group of flexible nucleotides.^3^ This information can then be used to improve the performance of RNA structure prediction methods (see, e.g., refs (4−10)). Chemical probing of small RNA molecules is usually performed in conditions that lead to the single-hit kinetics regime, where a single adduction per RNA molecule is formed on average,^11^ so that the typical spacing between adducts is on the order of a few tens of nucleotides at least. However, it is important to note that adduction requires a prior reversible physical binding followed by an irreversible chemical reaction. Even when the number of adductions per RNA molecule can be empirically verified, this cannot rule out a larger number of physical binding events in the proximity of the adduction site, potentially altering RNA dynamics and influencing the adduction rate. These physical binding events can be considered as a form of small-molecule crowding.^12^ Possible cooperative or anticooperative effects (see Figure 1) might lead to unexpected concentration-dependent reactivities.

**Figure 2:**
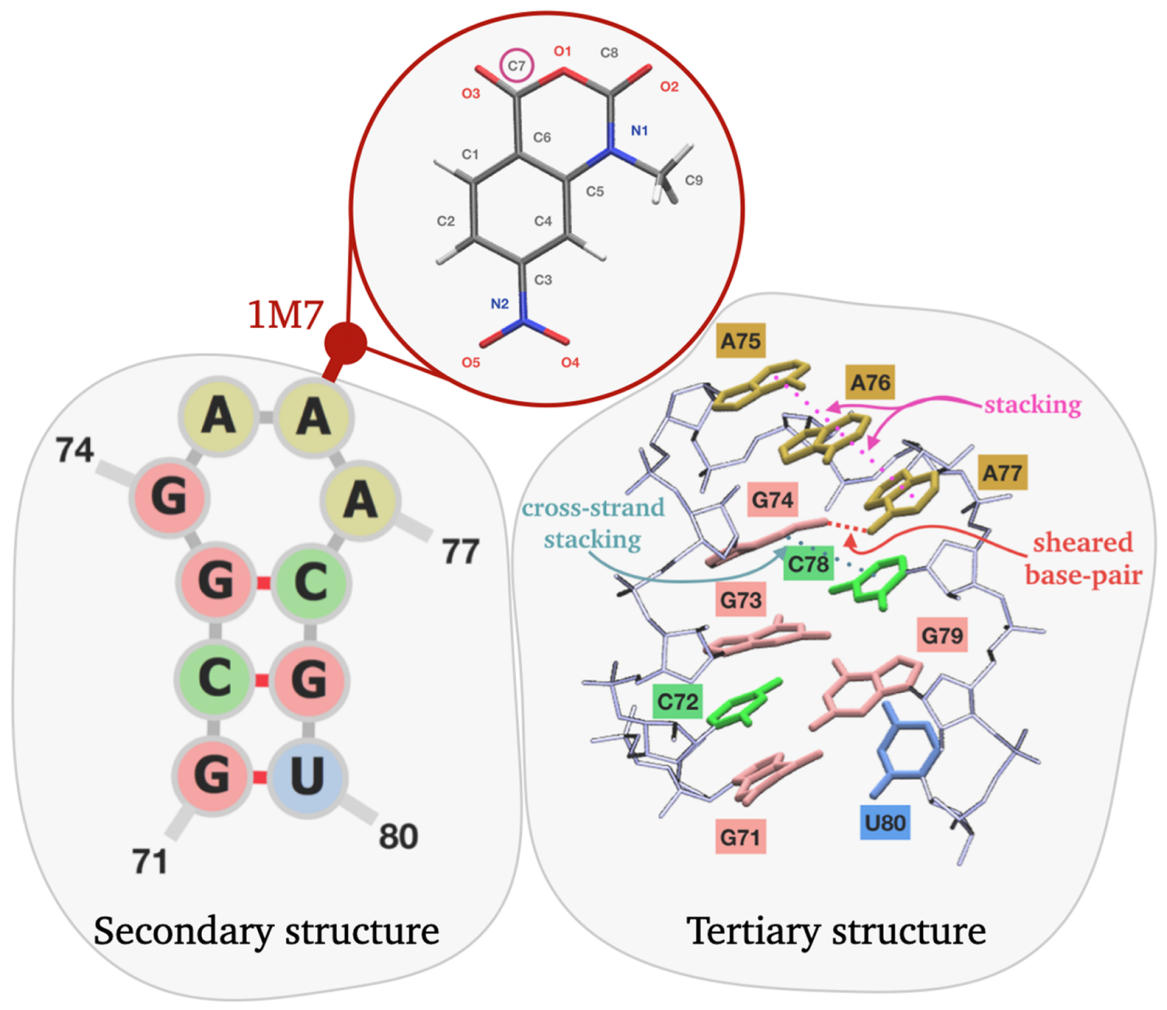
The gcgGAAAcgu tetraloop extracted from PDB 2GIS and the chemical probing reagent 1M7. Atom names of the parametrized 1M7 reagent are indicated, with the reactive site C7 circled in red. In the tertiary structure representation, nucleobases are shown as thick sticks and colored consistently with the secondary structure representation; noncanonical contacts are also highlighted.

Atomistic molecular dynamics (MD) simulations give direct access to RNA dynamics^13^ and have been used to characterize RNA flexibility and correlate it with SHAPE reactivity.^14−18^ In some of these works, MD simulations have been used to explicitly characterize the physical binding of SHAPE reagents to RNA in the infinite dilution limit, where a single reagent molecule is present.^16,18^ In principle, MD simulations with multiple copies of the reagent might help identifying (anti)cooperative effects at the typical experimental concentrations. In order to access concentration-dependent effects, however, one should perform simulations with unrealistically large boxes or, better, at constant chemical potential, where the number of copies of the reagent varies according to its concentration in a virtually infinite reservoir.^19^ Constant chemical potential simulations are usually performed using Monte Carlo techniques,^20^ which are inefficient if a bulky reagent (see Figure 2) is to be inserted in a condensed phase. These difficulties can be alleviated using an oscillating chemical potential,^21^ that however introduces some additional approximation, or using nonequilibrium candidate Monte Carlo.^22^ These Monte Carlo methods typically require specifically modified MD codes. Alternatively, a dedicated region of the box can be used as a reservoir, and a position-dependent potential can be added modulating the number of copies in the analyzed region using adaptive-resolution^23^ or constant-chemical-potential^24^ MD simulations. The adaptive-resolution method is not available in general purpose MD engines, whereas constant-chemical-potential MD is directly compatible with most simulation software via plugins such as PLUMED.^25^ However, both of these methods require parameters such as the size and shape of the transition and reservoir regions and the form of the bias potential to be chosen in advance.

In this work, we use MD simulations to investigate (anti)cooperative effects in the physical binding of a SHAPE reagent to a typical RNA structural motif. We introduce an approach to grand-canonical averaging that is based on a maximum-likelihood procedure used to analyze a set of simulations performed at a constant number of copies of the reagent molecule. The analysis requires the solution of a self-consistent set of equations similar to those employed in the weighted-histogram analysis method^26,27^ or in multistate Bennett acceptance ratio estimations.^28^ Importantly, the analysis is done as an *a posteriori* reweighting, so that it allows to choose and optimize the reservoir region after the simulations have been performed, computing weights to be associated with each of the simulated snapshots. In addition, the chemical potential or, equivalently, the concentration of reagent molecules in the buffer can be chosen *a posteriori*, thus allowing for the straightforward calculation of concentration-dependent properties from a single set of simulations. The method is then applied to compute the concentration-dependent physical binding affinity of a SHAPE reagent on an RNA tetraloop (see Figure 2). Interestingly, we predict that nucleotides in the loop undergo cooperative reagent binding at the typical experimental concentrations. Experimental data supporting the existence of cooperative effects in RNA tetraloops are also reported. Our observation opens the way to a new dimension in the interpretation of chemical probing data, where concentration-dependent results might be used to identify specific structural motifs.

## 2 Methods

### 2.1 Grand Canonical Reweighting of Molecular Dynamics

In order to describe physical situations where the number of particles is varying, the grand-canonical ensemble is necessary. In this ensemble, the fixed quantities are the chemical potential μ, that controls the fluctuations in the number of particles, the volume *V* of the system, and the temperature *T*. The ensemble can be represented as a canonical ensemble coupled to a particle reservoir that can gain or lose particles without appreciably changing μ. We consider the case where the chemical potential of a single species is controlled, whereas all the other species are simulated at a constant number of particles. We then introduce a procedure that can be used to obtain grand-canonical averages from the combination of a set  of *N*_max_ independent simulations, each with a different fixed number of those particles whose chemical potential is controlled, *N* ∈ {1, ..., *N*_max_}. The simulation box is divided into two subregions *A* and *B* that are assumed to be sufficiently decoupled. In the *N*th simulation, which is run in the canonical ensemble, the probability to observe *k* particles in region *A*/*B* is
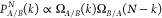
1Here, Ω_*A*_ and Ω_*B*_ are the canonical partition functions associated with regions *A* and *B*, respectively. We then define the count matrix **t** = {*t*_*Nk*_} which reports, for the trajectory with *N* copies of the particles, how many frames were seen with exactly *k* particles in region *A* and *N* – *k* particles in region *B*. We notice that this is a triangular matrix, since cases where *k* > *N* are impossible by construction. The probability to observe such a matrix can be computed as the probability to generate each of the corresponding frames and is equal to
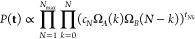
2Here, the normalization coefficients {*c*_*N*_} are required to ensure that, at fixed *N*, the sum of the probabilities *P*_*A*/*B*_^*N*^(*k*) over *k* is equal to one. The maximum-likelihood (ML) estimation of Ω_*A*_ and Ω_*B*_ is obtained by minimizing the negative log-likelihood −log  *P*(**t**). By using the Lagrange multiplier methods to include the normalization constraint mentioned above, one obtains the following Lagrangian function
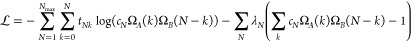
3where {λ_*N*_} is *N*_max_ Lagrangian multipliers. The notation can be simplified by defining the following: *A*_*k*_ = *∑*_*N*_*t*_*Nk*_, counting the number of times that, in the whole set of *N*_max_ trajectories, a particle was found in region *A*; *B*_*k*_ = *∑*_*N*_*t*_*N*,*N*–*k*_, counting the equivalent number for region *B*; and *L*_*N*_ = *∑*_*k*_*t*_*Nk*_, the total number of frames accumulated in the trajectory with *N* particles. In Section S1, we show that minimizing eq 3 as a function of Ω_*A*_ and Ω_*B*_ leads to the following coupled equations:
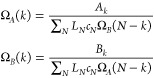
4These equations can be solved iteratively through the procedure reported in Algorithm S1. Since we made no assumption on the length of each trajectory {*L*_*N*_}, the method can be straightforwardly used also when the minimum number of simulated copies of the controlled particles is greater than 1 or when some simulations are missing, by just setting some of the elements of *L*_*N*_ to zero. Once the ML estimates of Ω_*A*/*B*_ have been obtained, they can be directly plugged in the grand-canonical probability of observing molecules in regions *A*/*B*, which is defined as
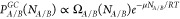
5This expression can be then used to compute the grand-canonical average of the number of particles in both regions *A* and *B*, at a fixed value of chemical potential μ.
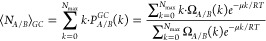
6Eq 6 provides a connection between the concentration in the experimental buffer and the chemical potential μ. Specifically, one can use the bisection method reported in Algorithm S2 to obtain μ corresponding to the desired concentration in region *B* (see Section S2).

Once μ has been obtained, grand-canonical averages in region *A* can be obtained by weighting frame *i* with a factor
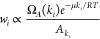
7where *k*_*i*_ is the number of copies of the molecule in region *A* in that frame. These weights, and the relationship between the concentration in region *B* and the chemical potential μ (eq 6), can be used to obtain arbitrary grand-canonical ensemble averages as smooth continuous functions of the concentration.

In short, our method is composed of the following steps: (a) a number of simulations are performed with a different number of copies of the particles and concatenated; (b) histograms counting how many times particles are present in regions *A* and *B* are computed; (c) these histograms are used to compute the canonical partition functions Ω_*A*_ and Ω_*B*_; (d) Ω_*B*_ is used to calculate which is the chemical potential μ corresponding to a given concentration; (e) μ and Ω_*A*_ are used to compute the weight associated with each of the frames of the initial concatenated trajectory.

### 2.2 Lattice Model

We test the method on a lattice space divided in two regions, *A* and *B*, containing *S*_*A*_ and *S*_*B*_ sites, respectively. Sites are then populated with a number up to *N*_max_ of particles that interact only through mutual exclusion: a site cannot be occupied by more than one particle. Two scenarios are tested: a purely entropic lattice, in which the free energy depends only on the number of possible configurations of the particles occupying the *S* = *S*_*A*_ + *S*_*B*_ site, and a lattice with one stabilizing site in region *A* that brings in a negative contribution to the free energy. The latter is supposed to mimic the situation where reagent molecules can bind to an RNA molecule that is located in region *A*. For more details, see Section S3.

### 2.3 GAAA Tetraloop of SAM-I Riboswitch

We then apply the introduced method to an RNA GNRA tetraloop (here N is any nucleotide and R is G or A), as this type of structural motif has some well-established properties: it presents (a) a highly stable secondary structure^29^ along with (b) rich dynamics involving multiple noncanonical contacts,^30,31^ that could lead to significant structural changes when in contact with SHAPE reagents; noticeably, (c) in SHAPE experiments, the GNRA tetraloop presents a typical reactivity pattern.^16^ We simulate a single loop motif rather than duplexes or larger structures in order to keep computational costs low, under the hypothesis that long-range effects are negligible. We expect this hypothesis to be reasonable as there is no evidence of conformational rearrangements due to interaction with SHAPE reagents, rather than at a local scale.^16^ The gcgGAAAcgu tetraloop is taken from the annotated structure of SAM-I riboswitch, that can be found in the PDB entry 2GIS.^32^ A representation of the resulting construct is shown in Figure 2. The stretch obtained in this way consists in a sequence of three base pairs, namely G71-U80, C72-G79, and G73-C78, plus the tetraloop under study: G74-A-A-A77. The initial conformation for this molecule is obtained by extracting the coordinates of the corresponding atoms from the PDB 2GIS entry. The closing base-pair of the sequence (G71-U80) is observed to unpair in preliminary simulations where a larger number of reagents is used. Since the calculation is meant to be representative of a GNRA tetraloop embedded in a longer RNA molecule, a harmonic restraint is applied to the hydrogen bonds between 71G/O6 and 80U/N3 and between 71G/N1 and 80U/O2, and these bases are excluded from analysis of reactivity and cooperativity to minimize terminal effects. RNA is parametrized according to the χOL3 version of the AMBER force field.^33−35^ Whereas this force field has known limitations, it has been shown to lead to reasonable stability and conformational dynamics for GNRA tetraloop motifs when simulations are initiated in the native state.^36^

### 2.4 Parametrization of 1-Methyl-7-nitroisatoic Anhydride (1M7)

1M7 is an efficient reagent used for SHAPE probing.^37^ The molecule is parametrized according to the general Amber force field (GAFF)^38,39^ for organic molecules using the Antechamber and parmchk tools implemented in Ambertools.^40^ The 1M7 probe structure is generated through the Maestro interface of the Schrödinger suite.^41^ The Gaussian 16 package is then employed for geometrical optimization and calculation of the electrostatic potential of the probe, using the B3LYP hybrid functional method with the 6-31G* basis set. Partial charges are then calculated using the RESP method^42^ as implemented in Antechamber. The resulting charges, that sum up to 0 as 1M7 is overall neutral, are reported in Table S1. The resulting Amber potential is then converted to the GROMACS implementation,^43^ using acpype.^44^ The optimized structure of 1M7 is reported in Figure 2.

### 2.5 Simulation Protocol

In order to sample a range of different concentrations of 1M7, *N*_max_ = 19 independent simulations are set up, each featuring a fixed number of probes, from *N* = 1 to *N* = *N*_max_. For each of them, the center of mass of the tetraloop is taken as origin of the reference frame. A rhombic dodecahedron simulation box is placed at a distance of 3 nm from the tetraloop. It is important to place the box at this step, before inserting the 1M7 probes, in order to preserve the volume across the simulations with different *N*’s. Reagents are placed at random points at equal distance from the tetraloop and with random orientation. In particular, the first probe is placed at a random point on the surface of a sphere, centered on the tetraloop and with a radius equal to the radius of gyration of the tetraloop plus 2 nm. The probe is then rotated about its center of mass by a random angle. A check on the distances between every atom pair is made in order to avoid clashes: if one of the atoms of the inserted probe is at a distance lower than 5 Å from any other atom, the insertion is rejected, and another point and orientation are generated. For each of the remaining *N* – 1 probes, the insertion procedure is repeated. Examples of the resulting conformation are represented in Figure S5 for *N* = 5 and *N* = 16. The resulting complexes are solvated using the OPC water model,^45^ and sodium counterions are added to neutralize the system.^46^ We preferred not to include a MgCl_2_ buffer, which is usually used in experiments, since the presence of divalent cations with strong binding might significantly slow down dynamics and require additional enhanced sampling protocols.^47^ In addition, the studied structural motif is known to be stable also in MD simulations performed in the absence of Mg^2+^ ions.^36^ The possible effects of the lack of negative ions in our main setup was instead explicitly checked in control simulations reported in the Results section. For each complex, the potential energy is minimized in order to relax the structures and remove possible clashes and incorrect geometries, through 50000 steps of the steepest descent algorithm. The minimization is followed by NVT equilibration of 1 ns up to a temperature *T* = 300 K and NPT equilibration at the same temperature, pressure *P* = 1 bar for another 1 ns using a Parrinello–Rahman barostat.^48^ A cutoff of 10 Å and the particle-mesh Ewald (PME) method^49^ are used for computing short-range interactions and long-range interactions, respectively. Temperature is controlled using the stochastic velocity rescaling thermostat.^50^ Equilibration is run with a time step of 2 fs with bonds involving hydrogens constrained via the LINCS algorithm.^51^ Production runs are then carried out in the NPT ensemble at *T* = 300 K and *P* = 1 bar. Plain MD simulations are performed using version 2018.5 of the GROMACS software.^43^

### 2.6 Statistical Uncertainties

To compute statistical uncertainties, we rely on a Bayesian bootstrap procedure^52^ where each entire trajectory is treated as a single data point. Specifically, at each bootstrap iteration (total *N* = 10000 iterations), we extract 19 weights from a Dirichlet distribution and use the resulting weighted trajectories to (a) estimate the canonical partition functions Ω_*A*_ and Ω_*B*_, (b) compute the chemical potential μ corresponding to the desired concentration in region *B*, and (c) use the resulting weights to compute the observable of interest. Given that trajectories are independent of each other, and at variance with standard block analysis,^53^ this estimate of the uncertainty is not subject to errors due to correlation between data points.

### 2.7 Experimental Methods

The DNA template corresponding to the GNRA tetraloop containing RNAs used in this study (PDB entries 2N2O,^54^2GIS,^32^2L1V,^55^1KXK,^56^1SCL,^57^1CQ5,^58^ and 2GV4(59)) with 5′ and 3′ SHAPE cassettes^60^ and the T7 promoter sequence was ordered from Eurofins Genomics. The RNA was transcribed and purified as previously described.^7^ SHAPE experiments were carried out with the 1M7 reagent at three final concentrations (3.2 mM, 6.5 mM, and 12.5 mM), and subsequent analysis of the concentration series was carried out as previously described.^7^ In particular, SHAPE modification was followed by reverse transcription, and resulting cDNA fragments were precipitated, redissolved, and separated using capillary electrophoresis. The normalization was carried out in a slightly modified way to enable the comparison of reads obtained at different concentrations. Specifically, reads were first normalized independently by dividing them by the sum of reads in the corresponding channel, with negative values replaced with zeros. Subsequently, the number of reads was multiplied by the nominal concentration. This step enforced a linear dependence of the average observed reads on the reagent concentration but importantly preserved the information about the position- and concentration-dependence of the reactivity.

## 3 Results

### 3.1 Lattice Model

In order to highlight the potential limitations of the simulation and reweighting protocol, we used our method to reconstruct the grand-canonical distributions for a lattice model. Results are presented in the Supporting Information, Section S3, and highlight the main limitation of the method, namely the fact that only concentrations that correspond to the number of particles in the set of analyzed simulations can be correctly reproduced. In addition, the model can be used to study the impact of statistical sampling errors on the estimated distributions.

### 3.2 Molecular Dynamics Simulations of SHAPE Reagents

In order to estimate the reactivity profile and cooperativity matrix of the gcgGAAAcgu tetraloop at different concentrations of 1M7, we first divide the simulation space into two regions: the binding region *A* is spherical, centered at the center of mass of the RNA motif with a fixed radius *r*_*A*_; the rest of the simulation space is defined as the buffer region *B*. In the binding region, the reagent copies are in proximity of the tetraloop and can form a relatively stable bound state, preliminary to the formation of the covalent bond that is not modeled here. In the buffer region, there are no direct interactions between reagent copies and RNA, and the formation of a bound state is not possible, as *r*_*A*_ is beyond the range of distances for binding. *N*_max_ = 19 trajectories are collected, each featuring *N* reagent copies with *N*∈[1,...,*N*_max_]. Every trajectory contains 10^5^ frames, corresponding to a total simulation length of 1 μs per trajectory. From the entire set of trajectories, we compute the number of times that each pair of nucleotides *i* and *j* in the tetraloop are in one of four possible pairwise binding states: both unbound, both bound to two different reagent copies, or only one of the two nucleotides bound to a reagent copy. We define binding between a nucleotide and a reagent copy to occur whenever the following two conditions are satisfied: a) the nucleotide is the nearest one to the probe, and b) the distance between the nucleotide and the probe is less than a certain threshold. For both conditions, we measure the distance by considering the atoms involved in the chemical reaction, namely the O2′ atom of the nucleotide and the C7 atom of the reactive carbonyl of 1M7. We set this threshold to *r*_*th*_ = 3.5 Å, consistently with ref (16).

By accumulating statistics on the *N*_max_ trajectories and using the introduced grand-canonical reweighting, we can estimate the partition functions Ω_*A*_ and Ω_*B*_, the value of the chemical potential μ that corresponds to the target reagent concentration, and the probability for one or two nucleotides to be bound to a reagent in the grand-canonical ensemble. Typically, SHAPE experiments using 1M7 as a probe are carried out at reagent concentrations ranging from 0.1 to 100 mM.^37^ Using a dodecahedral simulation box of volume *V*_*box*_ ≈ 439 nm^3^, the radius of the binding region fixed at *r*_*A*_ = 3 nm and *N*_max_ = 19 maximum number of reagent copies in the collected trajectories, the volume of the buffer region is *V*_*B*_ = *V*_*box*_ – 4/3*πr*_*A*_^3^ ≈ 316 nm^3^. With these settings, we can only reproduce reagent concentrations that are below a threshold of approximately  70 mM. Given μ and Ω_*A*_, one can obtain the weights for computing averages in the grand-canonical ensemble, which are denoted as *w*(*N*_*A*_) since, for each frame, the weight only depends on the number of copies of the reagent seen in region *A*.

### 3.3 Concentration-Dependent Reactivities

**Figure 3:**
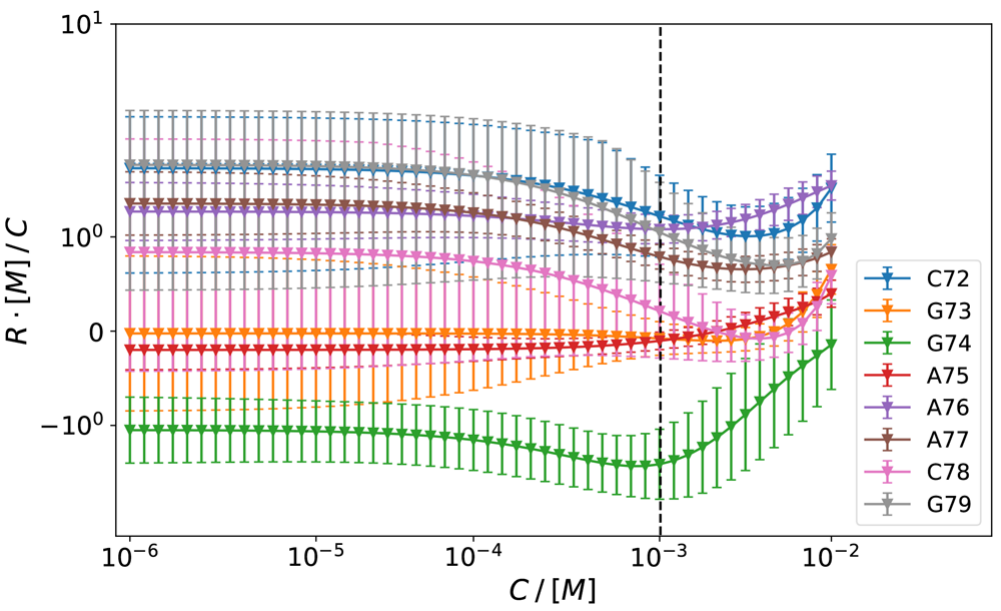
Reactivity, computed as the probability of each nucleotide to be physically bound to a SHAPE reagent, shown as a function of reagent concentration. Concentrations are reported in molar units, and the vertical dashed line denotes a typical lower bound for experimental concentrations (1 mM).

For each nucleotide, we estimate the reactivity *R*_*i*_ from the frequency with which it is observed in a bound state with any one of the reagent copies, and we average it in the grand-canonical ensemble, using the weights *w*(*N*_*A*_). In theory, the relation between the reactivity of a nucleotide and the concentration of reagent can be decomposed in the sum of terms representing the effect of a) the number of available reagent copies, b) the effect of a bound nucleotide on the binding probability of another nucleotide, due to (positive or negative) cooperativity, and c) higher order relations involving more than two nucleotides. The first term is proportional to the concentration, while higher-order terms depend on higher powers of the concentration. The dependency on reagent concentration of the binding affinities that we obtain for the simulated RNA motif is represented in Figure 3. At sufficiently low concentrations, the ratio *R*/*C* between reactivity and concentration saturates to a constant, consistently with the expected linear relationship. As reagent concentration is increased, and specifically for *C* > 10^–3^ M, higher-order contributions start to emerge significantly, as some reactivities show a nonlinear dependency on concentration. In principle, one nucleotide can exhibit positive cooperativity with some nucleotides and negative cooperativity with others. We attribute a superlinear relation between reactivity and reagent concentration to predominantly cooperative behavior, as binding affinity increases more than proportionally to the number of reagent copies available. As well, we interpret a sublinear dependency as a signal of predominantly anticooperative behavior and an approximately linear dependency either as the absence of cooperative effects or as positive and negative cooperativity behaviors compensating each other. Noticeably, the range of concentrations (10^–3^ to 10^–2^ M) that we identify as affected by cooperative effects overlaps significantly with the range of concentrations typically adopted in experiments. In this range, we quantify the nonlinearity of reactivity as a function of concentration for each nucleotide, by fitting power laws *R*_*i*_ = α · *C*^β^. Fit parameters are reported in Table 1. Uncertainties are computed here by performing the fitting at every bootstrap iteration and computing the standard deviation of the resulting coefficients. Although the statistical uncertainty on the individual points reported in Figure 3 is relatively high, errors associated with different values of the concentration are correlated, resulting in relatively low uncertainty in the estimated power coefficients (Table 1). In particular, for G74, we detect the strongest superlinear dependency. We notice that, with our definition of coefficients α and β, α corresponds to the reactivity extrapolated at a concentration of 1 M and thus is also affected by cooperative effects.

**Table 1:** Parameters of a Power Law of Reactivity *R* as a Function of Concentration *C*, Obtained by a Least-Squares Linear Fit of Their Logarithms, for Each of the Analyzed Nucleotides of the gcgGAAAcgu Tetraloop^a^

*R* = α · *C*^β^		
Nucleotide	β	α
C72	1.1 ± 0.2	11 ± 14
G73	1.3 ± 0.1	7 ± 6
G74	1.6 ± 0.2	19 ± 24
A75	1.2 ± 0.1	4 ± 2
A76	1.2 ± 0.1	13 ± 6
A77	1.0 ± 0.1	3 ± 1
C78	1.1 ± 0.2	4 ± 5
G79	0.9 ± 0.2	3 ± 4

a Powers β > 1 indicate cooperative behavior, while powers β < 1 indicate anticooperativity. Standard errors computed using bootstrap are reported.

### 3.4 Free-Energy Couplings

**Figure 4:**
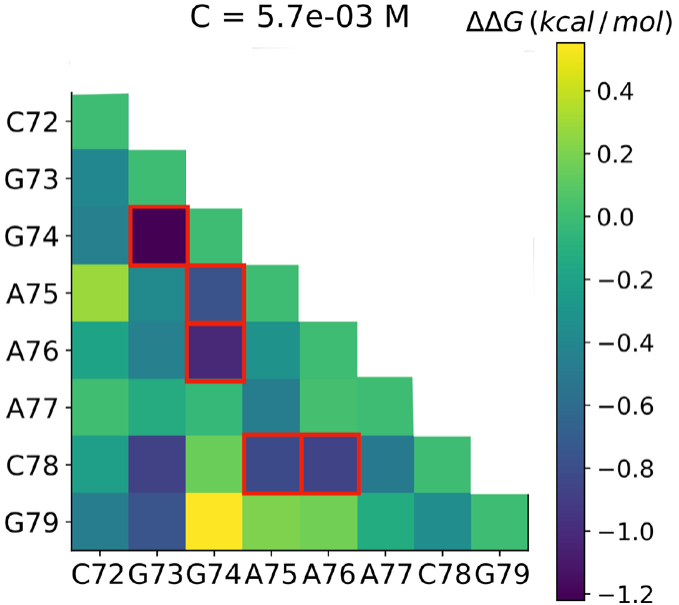
Cooperativity matrix ΔΔ*G* at typical reagent concentration. Pairs of nucleotides for which the cooperativity is different from zero with the significance level greater than 0.01 are highlighted in red. Anticooperative pairs with the significance level greater than 0.01 are not observed.

In order to quantify the cooperativity of nucleotides in reagent binding, we rely on the free-energy coupling model.^61^ Negative free-energy coupling ΔΔ*G*_*ij*_ < 0 means that the binding affinity of nucleotide *i* is increased if nucleotide *j* is bound to a reagent copy, so they are cooperative. Vice versa, positive ΔΔ*G*_*ij*_ > 0 means they are anticooperative. From the observed events, we can compute the frequency with which two nucleotides are in the same binding states and the frequency with which only one of the two is bound. From the ratio between these two frequencies, we compute the free-energy coupling for each pair of nucleotides, reweighted in the grand-canonical ensemble. The estimated values of ΔΔ*G* for an intermediate reagent concentration (*C* = 5.7 mM) among the tested ones are reported in Figure 4.

To identify pairs of nucleotides for which the cooperativity or anticooperativity is significantly different from zero, we check which fraction of the bootstrap samples returns a cooperativity or anticooperativity larger than zero. We set a significance level of α = 0.01. Since we deal with 28 hypotheses simultaneously, we rely on the Benjamini-Hochberg procedure^62^ to keep the false discovery rate of our estimates at level α. Pairs of nucleotides with significantly cooperative behavior are G73-G74, G74-A76, A76-C78, G74-A75, and A75-C78. At the same concentration, no significant anticooperative behavior is identified.

### 3.5 Conformational Dynamics

**Figure 5:**
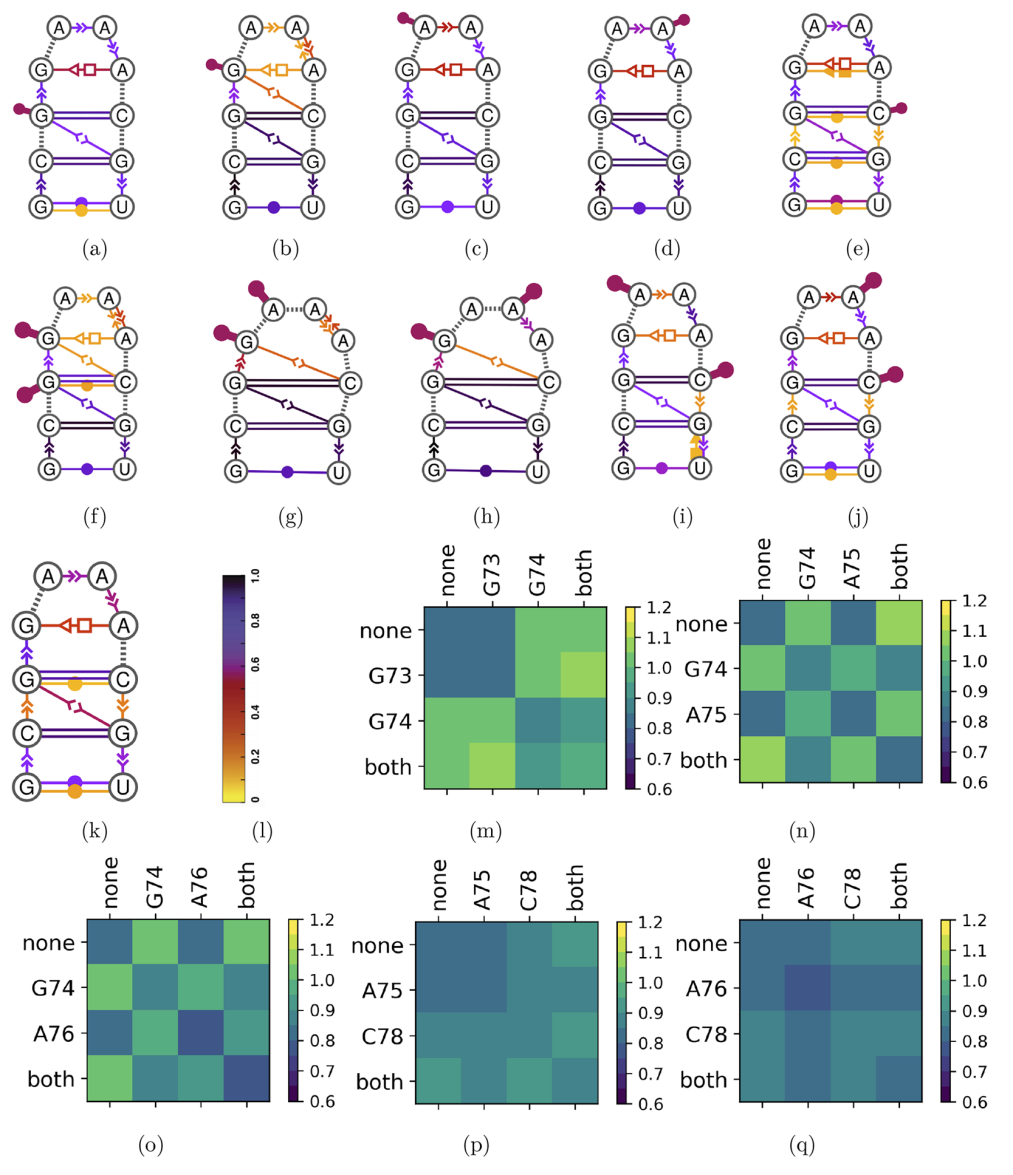
Conformational dynamics of the simulated RNA tetraloop under chemical probing with 1M7. Dynamic extended secondary structures representing base-pairing and base-stacking interactions following the Leontis-Westhof notation,^63,64^ for trajectories sampled under the constraint (a-e) that nucleotides in cooperative couples (a) G73, (b) G74, (c) A75, (d) A76, and (e) C78 are individually under probing by one of the reagent copies and (f-j) that the two nucleotides in each cooperative couple are simultaneously under probing. We performed a comparative analysis using as reference (k) a trajectory sampled under the constraint that no nucleotide is under probing by any of the reagent copies. The ensemble population of each interaction represented is reported (l) in the colormap. For each couple of cooperative nucleotides, we computed (m-q) the matrix of root-mean-square eRMSD deviations between the trajectories sampled with all the different constraints. Tick labels of the heatmaps indicate either which one of the two nucleotides is under the probing constraint or “none” and “both” respectively for the constraint of no nucleotide being probed and for both nucleotides being probed.

**Figure 6:**
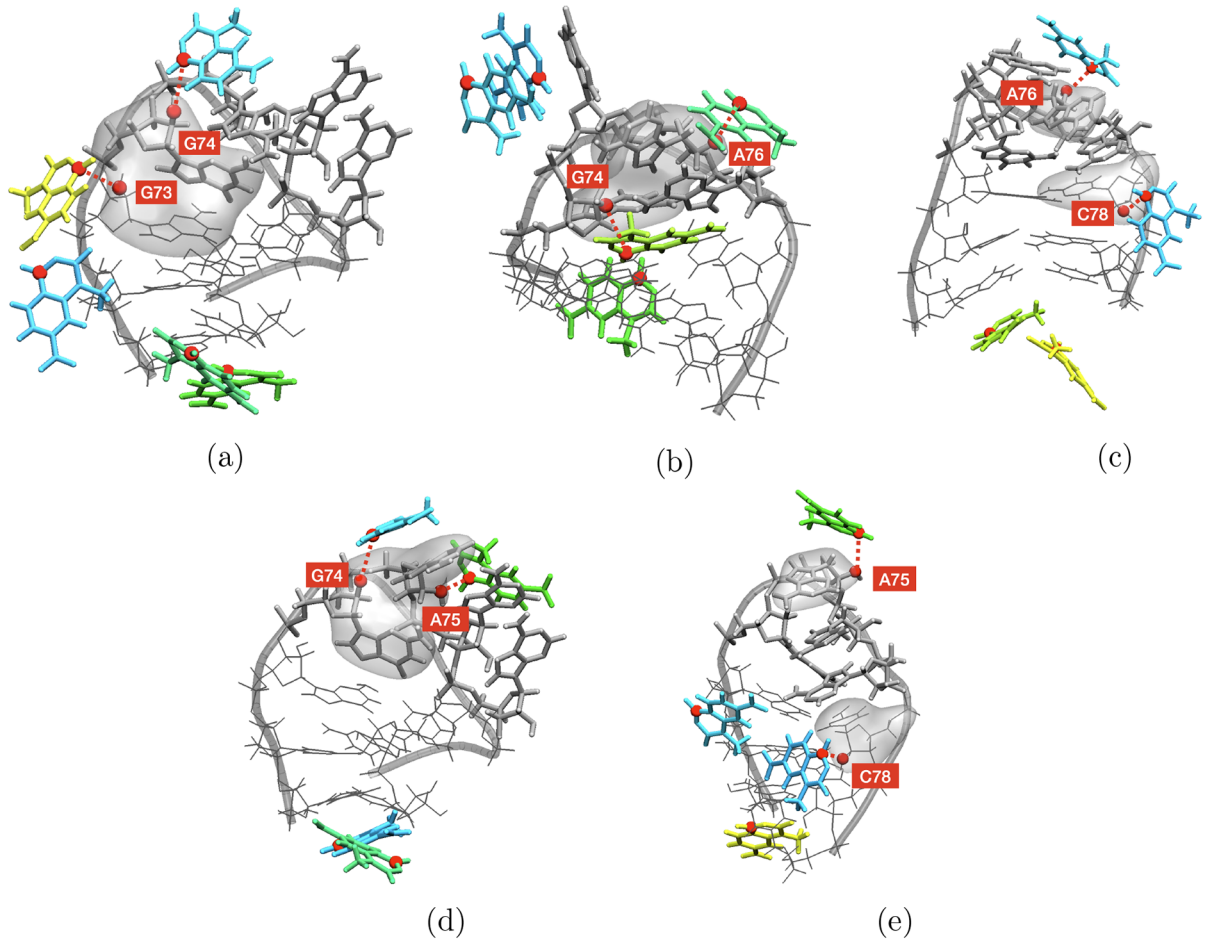
Representative selection of recurrent conformations of the tetraloop under simultaneous probing of cooperative nucleotides. The represented pairs are (a) G73 and G74, (b) G74 and A76, (c) A76 and C78, (d) G74 and A75, and (e) A75 and C78, consistently with Figure 5. Copies of the reagent are shown in colors; RNA atoms are shown in gray. Involved nucleotides are shadowed.

In order to investigate the structural signatures of cooperativity, we analyzed the conformations generated in all the simulated trajectories. In particular, we extracted sets of frames corresponding to specific conditions and analyzed them using the barnaba package,^64^ which allowed us to compute the eRMSD deviation^65^ between 3D structures and to show their dynamic extended secondary structure representation. In this representation, base-stacking and base-pairing interactions between nucleotides are reported according to Leontis-Westhof classification,^63^ and the frequency of each interaction is shown by coloring it according to the reported color map. Before analysis, frames were subsampled with weights *w*(*N*_*A*_) corresponding to their population at the intermediate concentration *C* = 5.7 mM. We first explored the possibility that conformational dynamics may be affected by reagent concentration. We thus performed an analysis of deviation, in terms of eRMSD, from the reference native structure of the structural ensembles sampled with the constraint that no nucleotide is under probing, i.e., with no reagent copy present in its binding region, as a function of reagent concentration. Results, that we reported in detail in the Supporting Information, Section S6, show that the presence of chemical probes in the neighborhood of the RNA molecule may affect its structure at sufficiently large concentrations, at which we observed cooperative effects, even when no probe is actually in the binding region of any nucleotide. In the following, we report a comparative analysis we performed at the level of conformational dynamics for trajectory frames in which the nucleotides that showed significant cooperativity effects are under probing. As a reference, we used a trajectory sampled with the constraint that no nucleotide is under probing. Results are shown in Figure 5. The computed eRMSD distances and the corresponding representations of the extended secondary structure in other settings we do not discuss here in detail are reported in Supporting Information, Section S7. For each cooperative couple of nucleotides, we computed the root-mean-square eRMSD distance between all pairs of frames from trajectories in which either one nucleotide or the other only are under probing, those in which the two nucleotides are simultaneously in the binding state, and the reference configuration in which no probe is in the binding region of any nucleotide (Figure 5k). From this reference calculation, we find a reference value (*eRMSD*_*none*,*none*_ = 0.84) which quantifies the natural fluctuations expected for this structural motif. Comparing the eRMSD between the ensembles constrained to G73, G74, none, and both being under probing (Figure 5a, b, f, k, and m) shows that the ensemble sampled when probing G73 is the closest to the reference (*eRMSD*_*G*73,*none*_ = 0.82) and has comparable heterogeneity (*eRMSD*_*G*73,*G*73_ = 0.81 and *eRMSD*_*none*,*none*_ = 0.84). Consistently, nucleotide G73 is observed to be probed from structures in which all the stable base-pairs of the reference ensemble are preserved, including the G74-A77 trans-Sugar/Hoogsten (tSH) base-pair. Conversely the ensemble sampled by probing G74 is the furthest from the reference (*eRMSD*_*G*74,*none*_ = 1.0), together with the one sampled when G73 and G74 are simultaneously probed (*eRMSD*_*both*,*none*_ = 1.1). The ensemble where G74 is bound displays a heterogeneity that is slightly larger than the reference state (*eRMSD*_*G*74,*G*74_ = 0.89). In this ensemble, the frequency of the G74-A77 tSH base-pair and of the inward A75-A76 stacking is reduced, whereas outward G74-C78 and inward A76-A77 stacking interactions emerge that are never observed in the reference. The structural ensemble sampled by the simultaneous probing of G73 and G74 is mostly affected by binding at position G74 (*eRMSD*_*both*,*G*74_ = 0.93 as compared to *eRMSD*_*both*,*none*_ = 1.1 and *eRMSD*_*both*,*G*73_ = 1.1), presenting the emergent G74-C78 and A76-A77 stacks at comparable (low) frequency as the G74-A77 tSH base-pair. The comparison is interestingly similar for the ensembles involving the couples G74-A75 (Figure 5b, c, g, and n) and G74-A76 (Figure 5b, d, h, and o). Differently from G73-G74 though, in the ensembles sampled by simultaneous probing of G74 and A75 and of G74 and A76, the tSH base-pair completely disappears. In the former, the inward A76-A77 stacking balances its upward equivalent observed in the reference, whereas in the latter, the reference one is preserved, resulting in a smaller distance (*eRMSD*_*both*,*none*_ = 1.0 for G74-A76 as compared to *eRMSD*_*both*,*none*_ = 1.1 for G74-A75). In all cases involving probing of one of the nucleotides from G73 to A77 (Figures S7, S8, S9, and S10) and in all the cases of simultaneous probing of G74 discussed so far, the C72-G73 upward stacking interaction is completely lost. These observations point to a possible major contribution of nucleotide G74 to cooperative effects. The presence of a probe in the binding region of G74 may indeed alter the conformational ensemble in such a way that stabilizing interactions in the loop are weakened and the binding affinity of other nucleotides in the loop is enhanced. The comparison between the relevant ensembles involving probing of nucleotide C78 (C78 alone under probing, C78 with A75 or A76 simultaneously under probing, Figure 5e, i, j, p, and q) shows smaller distances from the reference (*eRMSD*_*both*,*none*_ = 0.91 for A75-C78 and *eRMSD*_*both*,*none*_ = 0.88 for A76-C78). Together with the cooperativity measured from simulations, these observations suggest that specific structural motifs may be related to cooperative effects in probe binding, with the implication that real-world chemical probing experiments at sufficiently high concentration could result in concentration-dependent reactivities. In particular, the alteration of conformational ensembles as due to the presence of chemical probes may contribute the most when nucleotides close to the extrema of the loop motif are involved. Sample three-dimensional structures corresponding to simultaneous pairs of bound nucleotides are reported in Figure 6.

### 3.6 Importance of Grand-Canonical Reweighting

An important advantage of using the grand-canonical reweighting procedure introduced here with respect to simply consider the finite difference between simulations performed at a different number of particles is that smooth concentration-dependent curves can be extracted. In Section S8, we compare reactivities obtained at a fixed number of copies, obtained by separately analyzing some of the trajectories discussed above, and reactivities obtained at fixed chemical potentials, obtained by averaging over the entire concatenated trajectory. Behavior as a function of the chemical potential is visibly smoother than behavior as a function of the number of particles, thus making it easier to extract cooperative effects.

### 3.7 Control Simulations

We investigated the robustness of our results with respect to two properties of the simulation settings: helix length and ionic conditions. Since a systematic study of the effects of these properties on binding cooperativity is outside the scope of this work, we only generated two control trajectories, at a fixed representative value of the number of reagent copies, *N* = 6, and checked that reactivity profiles were consistent with those obtained from our study. In one case, we simulated a longer part of the SAM-I riboswitch, ranging namely from C69 to G82 (two additional base-pairs); in the other case, we increased the ionic strength. Comparisons with the main simulations are reported respectively in Section S9 and Section S10.

### 3.8 Comparison with Experimental Analysis

To validate our hypothesis of cooperative effects in the binding process of SHAPE reagents on RNA, we analyzed a limited number of experimental data sets. To the best of our knowledge, chemical probing databases only report results at one concentration (see, e.g., ref (66)). We thus generated our own data set. This analysis is limited to a small number of RNA structures and reagent concentrations but suggests that the effect is experimentally detectable. We considered a set of SHAPE experiments performed at three different reagent concentrations (3.2 mM, 6.5 mM, and 12.5 mM) for a set of 5 molecules for which reference crystallographic or NMR structures are available and can be used to identify the position of GNRA tetraloops (2GIS, 2 GAAA loops, 1KXK, 1 GAAA loop, 1SCL, 1 GAGA loop, 1CQ5, 1 GGAA loop, and 2GV4, 1 GAAA loop) and 2 molecules not containing GNRA tetraloops (2N2O and 2L1V). These concentrations are similar to those used in other works (see, e.g., ref (67)) and are in the range in which our simulations predict cooperative effects (see Figure 3). We then performed an analysis equivalent to the one reported in Section 3.3 to obtain an exponent β and a scaling factor α associated with each nucleotide. Additionally, we performed a Student’s *t*-test to compare the distributions of β and α between the two groups: nucleotides located in the GNRA loops (GNRA) as compared to other nucleotides (Other). It is important to recall that, with our definition of coefficients α and β (see Table 1), α corresponds to the reactivity extrapolated at a concentration of 1 M. This value is highly sensitive to experimental errors on β. We thus decided to show , which corresponds to the reactivity shifted to 0.0125 M. This value is close to the reactivity measured at 0.0125 M but more robust since it includes information from multiple experiments.

**Figure 7:**
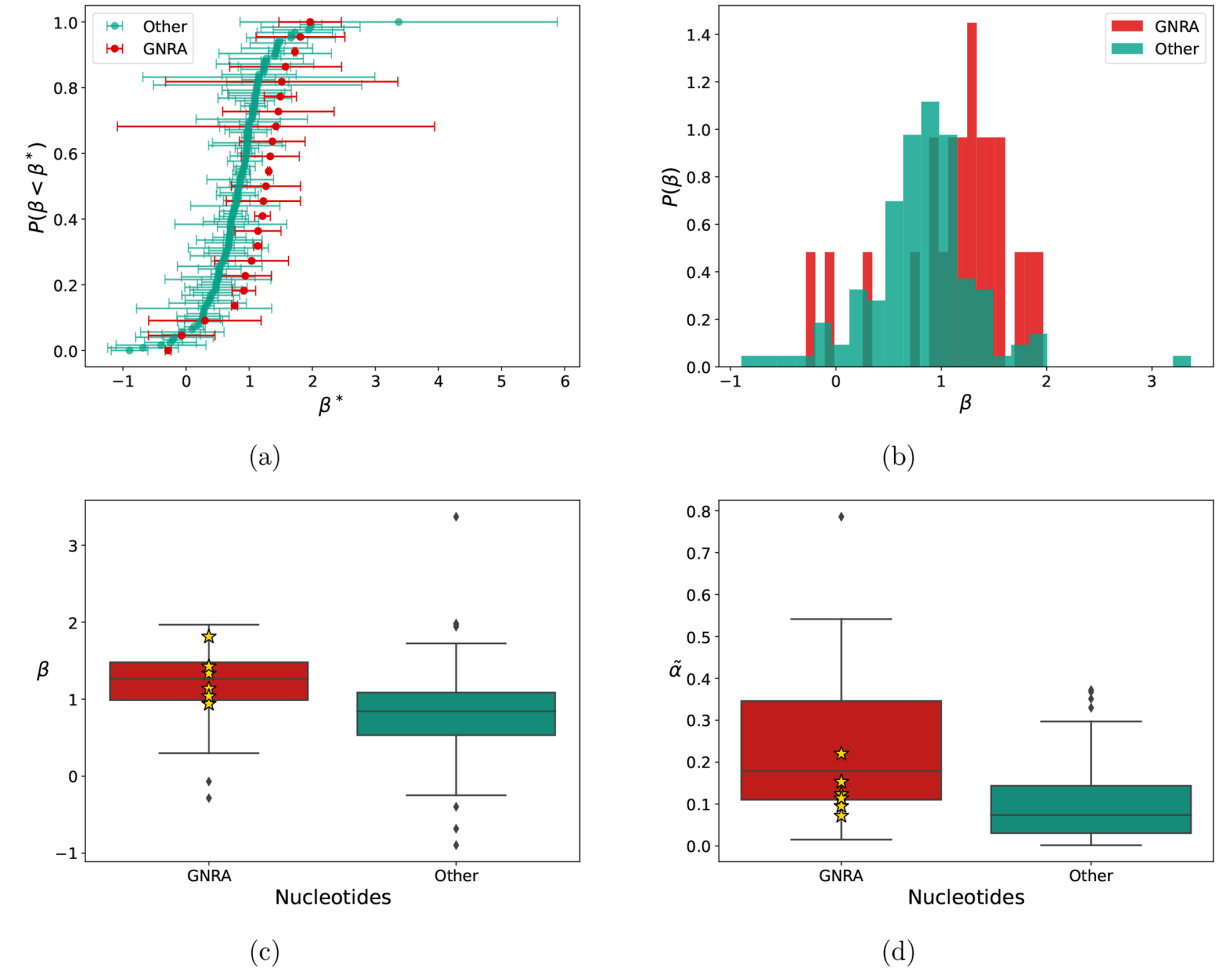
Analysis of cooperativity over a set of experimental structures. SHAPE reactivities of nucleotides in the GNRA loop (GNRA) and of other nucleotides (Other) are measured at three different concentrations (3.2 mM, 6.5 mM, and 12.5 mM), and power-law fitting is carried out as in Section 3.3. Cumulative distribution and histograms (a-b) for β show a significant difference between GNRA and other nucleotides. Panel (a) also shows the standard error of the linear fit. Box plots for both β and  are also shown (c-d). Stars indicate the value for the Gs located at the initial position of GNRA tetraloops.

As shown in Figure 7(a-c), the values of β for nucleotides located in GNRA loops are significantly larger than those for other nucleotides (p-value *p* = 0.006, *t* test for the means). In particular, we observe an average value of the exponent greater than 1 for GNRA nucleotides (β_*GNRA*_ = 1.16). Cooperativity obtained by averaging only on G nucleotides located in the first position of the tetraloops is even larger (β_*G*1_ = 1.28). Conversely, for other nucleotides, the average is lower than 1 (β_*other*_ = 0.84). These observations support the fact that nucleotides in GNRA loops might be affected by cooperative binding effects, more likely than other nucleotides, and that the cooperativity is larger at position 1 in the tetraloops.

We also observe significantly larger values of α for GNRA nucleotides (, , p-value *p* < 10^–4^, *t* test for the means). This is expected since it is related to the usual paradigm of nonpaired nucleotides being less reactive than paired nucleotides. Interestingly, the average value for G nucleotides located in the first position of the tetraloops is significantly lower . The low reactivity of the first G in a GNRA tetraloop has been also found in a previous work,^16^ both analyzing previously published experimental data^68^ and running MD simulations, although using a different SHAPE reagent.

## 4 Discussion

In this work, we use molecular dynamics simulations to identify possible cooperative mechanisms in the binding process of SHAPE reagents on RNA. We first develop a method to obtain concentration-dependent averages in the grand-canonical ensemble by combining simulations done with a different number of copies of the reagent. We show how the method works on a lattice model. Finally, we use it to analyze simulations of 1M7 reagents interacting with a typical RNA structural motif. Example Python notebooks that can be used to repeat our calculations are available on GitHub at https://github.com/bussilab/shape-grandcanonical-md. Complete trajectories have been uploaded on Zenodo at 10.5281/zenodo.7139540.

The introduced method is based on an idea similar to the one used in weighted-histogram analysis,^26,27^ where a maximum likelihood procedure is used to combine statistics obtained simulating a different number of copies of the reagent molecule. We derived and tested our analysis protocol so as to control the chemical potential of a single molecular species. However, the formalism could be easily extended to multiple molecular species, at the price of setting up a multidimensional grid of simulations where the number of copies of each species is scanned. With respect to the straightforward comparison of simulations performed at different number of particles, our method has the advantage that it allows to compute properties as smooth functions of the chemical potential and, thus, of the particle concentration.

At variance with methods based on grand-canonical Monte Carlo^21,22^ or position-dependent potentials,^23,24^ the introduced procedure only requires analyzing plain MD simulations. This means that any MD code could be used and that there will be no overhead associated with changing on-the-fly the number of copies of each molecule in the simulation box or to compute thermodynamic forces to control the number of copies in a given region. However, this advantage comes at a price. If the actual concentration of the species is unknown, it might be difficult to set up an appropriate range for the number of copies of molecule to be included in each simulation. Since results will only be reliable for concentrations that have been actually sampled, this might lead to the need to perform further simulations with a different number of copies. However, also in this case, all simulations could be easily combined to maximize the statistical efficiency. Similarly to methods based on position-dependent potentials,^23,24^ our approach is based on the approximation that subregions of the simulation cell are sufficiently decoupled. This might limit its applicability in cases where interactions are long ranged, such as electrolyte solutions, unless ion concentrations are large enough to provide a significant screening and make interactions effectively short ranged.^69^ At variance with methods based on position-dependent potentials, however, our procedure allows the region to be selected in the analysis phase. This in principle allows for fine-tuning its definition *a posteriori*, without the need to repeat the MD simulations. An advantage of grand-canonical Monte Carlo methods is that they can be used to effectively enhance the conformational sampling of the controlled species, which could appear on both sides of a high free-energy barrier. For instance, this would increase sampling of hidden binding pockets. On the other hand, our method, and methods based on position-dependent potentials, should be explicitly combined with enhanced sampling methods to cross large free-energy barriers.^70^ Although in this work we only analyzed plain MD simulations, enhanced sampling simulations could be analyzed by considering the bias potential when computing the weighting factors.

The method was then applied to the characterization of the dynamics of an RNA structural motif interacting with SHAPE reagents at various concentrations. Results were obtained by combining 19 independent simulations with a different number of copies of the reagent. Statistical uncertainties were estimated using Bayesian bootstrapping over the 19 independent simulations. Since the simulations were prepared independently of each other, the trajectories can be considered as statistically independent. This procedure allowed us to define confidence intervals for all the examined quantities. We were then able to estimate the concentration-dependent probability with which each nucleotide can be bound to a SHAPE reagent. We also estimated cooperativity effects by analyzing all pairs of nucleotides, showing that pairs of positions located in the RNA tetraloop display a stronger cooperativity. This cooperativity can be explained as a combination of multiple factors, including interaction between copies of the reagent and induced changes in the RNA conformational ensemble. It is worth noting that we did not observe any pair of positions with a statistically significant anticooperativity. Based on our structural analysis, which shows that reagent binding leads to local RNA destabilization, this is expected. Inter-reagent stacking can also be reasonably expected to lead to cooperative rather than anticooperative effects. However, we cannot rule out that anticooperative effects might arise in more complex structural motifs where, for instance, physical binding in a position might result in a steric hindrance for binding in a neighboring position or even to larger conformational changes of the probed RNA. It is also important to observe that more complex structural patterns might make the cooperative or anticooperative effects nonlocal. For instance, the stability of tertiary contacts in the native structures might be impacted by probe concentration, due to probe-RNA interaction or possibly altered RNA structural dynamics. The study of these long-range interactions is nontrivial but could be addressed with either extensive MD simulations or with more systematic analysis of experimental data.

The fact that the presence of chemical probes can alter the RNA conformational ensemble is particularly relevant and deserves a separate discussion. In one sense, it might be argued that, if chemical probes alter the conformational ensembles, the experiment will report reactivities corresponding to conformations that are further from physiological conditions and possibly of lower biological interest. On the other hand, if these effects are predictable and it is possible to *a priori* ascribe them to specific structural motifs, the observed effects can be used to improve the capability of the experiment to detect those specific motifs. The current work is meant as a proof of concept in this sense, since more experimental data on a wider range of motifs would be required to enable a quantitative analysis of this effect.

Importantly, our work only addresses the physical binding between the reagent and RNA and completely neglects the subsequent acylation step. This is reasonable within the working hypothesis that physical binding gives the dominant contribution to the heterogeneity of the observed reactivities as a function of the nucleotide position and as a function of the reagent concentration. In other words, we assume that the acylation step provides an approximately uniform prefactor to the observed reactivities. In this sense, a quantitative comparison between the predicted and observed reactivities is not feasible, and we can only compare ratios between reactivities observed at different positions or at different reagent concentrations. A detailed modeling of the acylation step is out of the scope of this work. We argue that a proper quantum-mechanical calculation of the reaction would be difficult, and specifically, it might be challenging to reach the statistical accuracy necessary to observe differences between nucleotides in different structural environments. We speculate that quantum-mechanical calculations might be more useful to characterize the difference between different reagent molecules.

The observed cooperativity could be directly detected experimentally, by measuring nonlinearities in the dependence of the SHAPE reactivity on the reagent concentration. Experimental data collected for this work shows that a systematic effect can be observed where nucleotides located in GNRA tetraloops display a nonlinear dependence when compared to the average reactivity, in qualitative agreement with the results of our simulations. Whereas the analyzed set of experimental data is limited, this observation suggests that the effect might be general and could be tested with more systematic experiments performed on a range of reagent concentrations. The generation of more systematic experimental data points would be required to quantitatively validate our predictions. Particularly interesting could be the investigation of alternative structural motifs, such as tertiary contacts and pseudoknots. A technical but important issue that was not considered here is the fact that SHAPE reagents are being inactivated by water, resulting in an effective concentration of active reagents that might be lower than the nominal one. We are not aware of experimental estimates of the concentration of active reagents, and thus, we qualitatively used the nominal concentration as a proxy of the effective one. We additionally note that inactivated (hydrolyzed) reagents are negatively charged and thus are expected to be electrostatically repelled by RNA and less effective than active ones in the crowding effect that is investigated in this work.

An important outcome of this work is that it suggests that different structural motifs might have a different degree of cooperativity. In this sense, more information could be profitably extracted from experiments performed at different reagent concentrations. Many different approaches have been suggested to analyze SHAPE reactivities to improve RNA structure prediction, including the idea of identifying reactivity patterns for known motifs^9^ and of combining data obtained with different reagents.^5,8^ However, we are not aware of any attempt to use concentration-dependent information as it is suggested here. The measurement of concentration-dependent reactivities for a sufficiently large number of training RNA systems of known structure is left as a subject for a future work.

## Supporting Information Available

Derivation of the algorithm with likelihood maximization; relationship between chemical potential and concentration; results on a lattice model; parameters for 1M7; sample initial conformations; structural analysis at various concentrations; effects of grand-canonical reweighting, ionic conditions, and system size (PDF)
