# Supplementary material for: Molecular Dynamics Simulations with Grand-Canonical Reweighting Suggest Cooperativity Effects in RNA Structure Probing Experiments
Source: J Chem Theory Comput. 2023 Jun 8;19(12):3672–85. doi: 10.1021/acs.jctc.3c00084 (PMC10308816; doi:10.1021/acs.jctc.3c00084)
Supplement: Supplementary file 1 — ct3c00084_si_001.pdf [file ct3c00084_si_001.pdf]

Supporting Information for Molecular dynamics simulations with grand-canonical reweighting suggest cooperativity effects in RNA structure probing experiments

Nicola Calonaci,[†,‡] Mattia Bernetti,[†] Alisha Jones,[¶,§] Michael Sattler,[¶,∥] and Giovanni Bussi[*,†]

†*Scuola Internazionale Superiore di Studi Avanzati, SISSA, via Bonomea 265, Trieste 34136, Italy*

‡*Department of Mathematics and Geosciences, University of Trieste, Trieste 34127, Italy*

¶*Institute of Structural Biology, Helmoltz Zentrum Muünchen, Neuherberg 85764*

§*Bavarian NMR Center at Department of Chemistry, Technical University of Munich, Garching 85757, Germany*

∥*Center for Integrated Protein Science München and Bavarian NMR Center at Department of Chemistry, Technical University of Munich, Garching 85757, Germany*

E-mail: bussi@sissa.it

S1 Likelihood maximization

The likelihood of observing $\mathbf{t} = \{t_{Nk}\}$ frames from a set $\mathcal{S}$ of N_{max} independent simulations, with t_{Nk} frames with k particles in region A and $N-k$ particles in region B, is

$$P\left(\mathbf{t}|\Omega_A, \Omega_B, \{c_N\}\right) \propto \prod_{N=1}^{N_{max}} \prod_{k=0}^{N} \left(c_N \Omega_A(k) \Omega_B(N-k)\right)^{t_{Nk}} \tag{S.1}$$

up to a normalization constant. The normalization coefficients $\{c_N\}$ are required to ensure that, for each value of the number of reagent copies N

$$\sum_{k=0}^{N} c_N \Omega_A(k) \Omega_B(N-k) = 1 \tag{S.2}$$

Taking the negative logarithm of the likelihood and adding Lagrangian multipliers $\{\lambda_N\}$ to ensure the above normalization requirement yields

$$\begin{aligned}&\mathcal{L}\left(\mathbf{t}|\Omega_A, \Omega_B, \{c_N\}\right) = \\ &\quad -\sum_{N=1}^{N_{max}} \sum_{k=0}^{N} t_{Nk} \log\left(c_N \Omega_A(k) \Omega_B(N-k)\right) - \sum_{N} \lambda_N \left(\sum_{k} c_N \Omega_A(k) \Omega_B(N-k) - 1\right)\end{aligned} \tag{S.3}$$

The Lagrangian function $\mathcal{L}$ can be minimized by setting to zero its gradient with respect to the model parameters Ω_A, Ω_B, $\{c_N\}$, and to the Lagrangian multipliers $\{\lambda_N\}$. The resulting equations are:

$$\frac{\partial \mathcal{L}}{\partial \lambda_N} = \sum_{k} c_N \Omega_A(k) \Omega_B(N-k) - 1 = 0 \tag{S.4a}$$

$$\frac{\partial \mathcal{L}}{\partial c_N} = -\frac{\sum_k t_{Nk}}{c_N} - \lambda_N \sum_{k} \Omega_A(k) \Omega_B(N-k) = 0 \tag{S.4b}$$

$$\frac{\partial \mathcal{L}}{\partial \Omega_A(k)} = -\frac{\sum_N t_{Nk}}{\Omega_A(k)} - \sum_{N} \lambda_N c_N \Omega_B(N-k) = 0 \tag{S.4c}$$

$$\frac{\partial \mathcal{L}}{\partial \Omega_B(k)} = -\frac{\sum_N t_{N,N-k}}{\Omega_B(k)} - \sum_{N} \lambda_N c_N \Omega_A(N-k) = 0 \tag{S.4d}$$

From the equation S.4a, we obtain

$$\sum_k \Omega_A(k)\Omega_B(N-k) = \frac{1}{c_N} \tag{S.5}$$

that we replace in the second term of the equation S.4b yielding

$$\lambda_N = -\sum_k t_{Nk} \tag{S.6}$$

We then define: $A_k = \sum_N t_{Nk}$, counting the number of times that, in the whole set of N_{max} trajectories, a particle was found in region A; $B_k = \sum_N t_{N,N-k}$, counting the equivalent number for region B; and $L_N = \sum_k t_{Nk}$, the total number of frames accumulated in the trajectory with N particles. By substituting Eq. S.6 and the definitions of A_k, B_k, and L_N in Equations S.4c and S.4d we obtain

$$\begin{aligned} \Omega_A(k) &= \frac{A_k}{\sum_N L_N c_N \Omega_B(N-k)} \\ \Omega_B(k) &= \frac{B_k}{\sum_N L_N c_N \Omega_A(N-k)} \end{aligned} \tag{S.7}$$

These equations can be solved iteratively through the algorithm reported in Alg. S1. Noticeably, line 9 to 12 provide a normalization of Ω_A and Ω_B such that $\Omega_A\left(k=0\right)=1$ and $\Omega_B\left(k=0\right) = \Omega_B\left(k=1\right) = 1$. In this way the free energy of the state with no particle at all is set to zero, as well as the free-energy cost for adding the first particle to region B. Since the chemical potential μ is defined up to a constant, Ω_A and Ω_B are invariant with respect to scaling by an arbitrary factor f each, and to scaling each k-th component of $\Omega_{A/B}$ by the k-th power of the same factor f^k. By choosing $f = \Omega_B\left(k=1\right)/\Omega_B\left(k=0\right)$ the normalization is fixed and the scaling invariance is removed. This scaling has no impact on the final weights, but can affect the relationship between the chemical potential and the particle concentrations and can be used to assign a physical interpretation to the obtained canonical partition functions.

Algorithm S1 Estimating Ω_A and Ω_B

$\Omega_A^{i=0}[k] \leftarrow A[k] \quad \forall k \in \left[0, \ldots, N_{max}\right]$

$\Omega_B^{i=0}[k] \leftarrow B[k] \quad \forall k \in \left[0, \ldots, N_{max}\right]$

threshold $\leftarrow 10^{-30}$

for $i \in \{1, \ldots, N_{steps}\}$ **do**

    $c[N] \leftarrow 1 / \sum_{k=0}^{N} \Omega_A^{(i-1)}[k] \cdot \Omega_B^{(i-1)}[N-k] \quad \forall N \in \left[1, \ldots, N_{max}\right]$

    $\Omega_A^{(i)}[k] \leftarrow A[k] / \sum_{N=k}^{N_{max}} L[N] \cdot c[N] \cdot \Omega_B^{(i-1)}[N-k] \quad \forall k \in \left[0, \ldots, N_{max}\right]$

    $\Omega_B^{(i)}[k] \leftarrow B[k] / \sum_{N=k}^{N_{max}} L[N] \cdot c[N] \cdot \Omega_A^{(i-1)}[N-k] \quad \forall k \in \left[0, \ldots, N_{max}\right]$

    $\varepsilon \leftarrow \sum_{k=0}^{N_{max}} \left[\left(\Omega_A^{(i)}[k] - \Omega_A^{(i-1)}[k]\right)^2 + \left(\Omega_B^{(i)}[k] - \Omega_B^{(i-1)}[k]\right)^2 \right]$

    $\Omega_B^{(i)} \leftarrow \Omega_B^{(i)} / \Omega_B^{(i)}[0]$

    $f \leftarrow \Omega_B^{(i)}[1] / \Omega_B^{(i)}[0]$

    $\Omega_B^{(i)}[k] \leftarrow \Omega_B^{(i)}[k] / (f^k B[0]) \quad \forall k \in \left[0, \ldots, N_{max}\right]$

    $\Omega_A^{(i)}[k] \leftarrow \Omega_A^{(i)}[k] / (f^k A[0]) \quad \forall k \in \left[0, \ldots, N_{max}\right]$

    if $\varepsilon <$ threshold **then**

        break

S2 Fixing reagent concentration through the chemical potential

Once the ML estimates of Ω_A and Ω_B are computed, estimates of grand-canonical averages of any function $f(k)$ of the number of particles k in either region A or B can be computed as

$$\langle f(k) \rangle_{GC} = \sum_{k=0}^{N_{max}} f(k) \cdot P_{A/B}^{GC}(k) = \frac{\sum\limits_{k=0}^{N_{max}} f(k) \cdot \Omega_{A/B}(k) e^{-\mu k/RT}}{\sum\limits_{k=0}^{N_{max}} \Omega_{A/B}(k) e^{-\mu k/RT}} \tag{S.8}$$

where μ is the chemical potential. It is thus straightforward to compute the grand-canonical average of the number of reagent copies in both regions by replacing $f(k) = k$ in the equation above. Since we want to compute averages of quantites such as affinity and cooperativity at varying reagent concentration, we first have to identify the values of μ corresponding to the desired concentrations, using the algorithm reported in Alg. S2.

Algorithm S2 Estimating μ as function of the number of particles in B

function NB_OF_MU(μ)

    $OB \leftarrow \Omega_B$ obtained using Alg. S1

    $NB \leftarrow$ desired N_B

    $P_B[k] \leftarrow OB[k] \cdot e^{-\mu k/RT} \quad \forall k \in \left[0, \ldots, N_{max}\right]$

    $P_B \leftarrow P_B / \sum\limits_{k=0}^{N_{max}} P_B$

    $N_B^{est} \leftarrow \sum\limits_{k=0}^{N_{max}} k \cdot P_B[k]$

    return $\log N_B^{est} - \log NB$

find the root of NB_OF_MU through an optimized bisection routine

S3 Lattice model

The introduced methodology for grand-canonical reweighting of molecular dynamics presented in the Methods section in the main text is first tested on a lattice model. We consider a lattice space divided in two regions, A and B. Region A contains S_A sites and region B contains S_B sites. Sites are then populated with a varying number of particles that interact with each other only through mutual exclusion, so that a site cannot be occupied by more than one particle. We first consider two possible scenarios: a purely entropic systems, in which all the sites are equivalent and the free energy depends only on the entropic contribution of the number of possible combinations of up to N_{max} particles occupying the $S = S_A + S_B$ sites; and a system in which the presence of a stabilizing site in the lattice region A brings in an additional energetic contribution to the free energy. In both cases, the partition functions Ω_A of region A and Ω_B of region B can be computed analitically. These functions are normalized as explained in Section S1, that is by setting $\Omega_A(k = 0) = \Omega_B(k = 0) = 1$, and $\Omega_B(k = 1) = 1$, so that the zero of free energy corresponds to the empty lattice and the free-energy cost for insertion of the first particle in region B is set to zero. The normalization is accomplished by scaling each $\Omega_{A/B}(k)$ by a factor $1/(f^k \Omega_{A/B}(0))$, where $f = \left(\frac{\Omega_B(1)}{\Omega_B(0)}\right)$.

In the purely entropic system, the two partition functions are related to the number of different combinations in which particles can be distributed in the sites:

$$\Omega_{A/B}(k) \propto \binom{S_{A/B}}{k} = \frac{S_{A/B}!}{(S_{A/B} - k)!k!} \tag{S.9}$$

If the number of sites in A and B is equal, then populating a site in A has the same free-energy cost of populating one in B. The free-energy contributions of the two regions $F_{A/B} = -\log \Omega_{A/B}$ for numbers of sites $S_A = S_B = 20$ is shown in Fig. S1a.

In the second scenario, regions A and B differ for the presence of a single stabilizing site in A, for which the probability to be populated is 100 times larger than the other sites, which

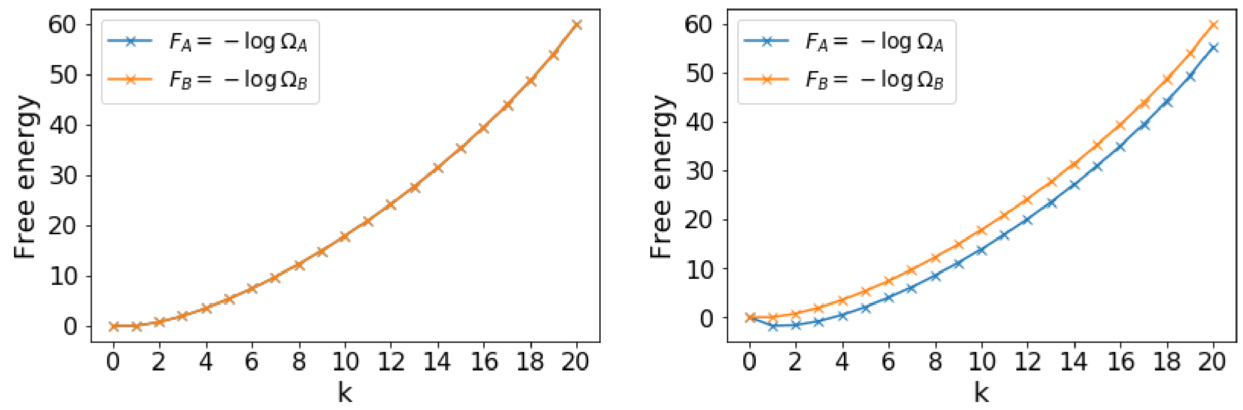

(a) (b)

Figure S1: Free-energy contributions of the two regions A and B of a lattice space populated with mutually exclusive particles. In a purely entropic system with A as large as B (panel a), the two lines are indistinguishable. In a system with a stabilizing site in region A (panel b), the free energy gain associate to insertion in region A rather than region B can be seen.

corresponds to a stabilization of $-RT \log 100$.

$$\Omega_A(k) \propto \binom{S_A - 1}{k} + 100 \binom{S_A - 1}{k - 1} \tag{S.10}$$

As shown in Fig. S1b, when at least one particle is present in the lattice, the presence of the stabilizing site systematically contributes with a free-energy gain ($-\log \Omega_A(k) < -\log \Omega_B(k)$). Since the second case is more representative of a molecular system where particles can interact with a a solute molecule in region A, we consider only this case for the following tests, but similar results could be obtained for the purely entropic system.

We first address the limitations of the method arising from the fact that a finite N_{max} is used. We assume to be able to collect the information about the probability of observing a given number of particles in region A or B from a set of simulations performed with a fixed number of particles ranging from 1 to N_{max}. These probabilities are $A_k \propto \sum_{N=1}^{N_{max}} \frac{\Omega_A(k)\Omega_B(N-k)}{\sum_{k'} \Omega_A(k')\Omega_B(N-k')}$ and $B_k \propto \sum_{N=1}^{N_{max}} \frac{\Omega_A(N-k)\Omega_B(k)}{\sum_{k'} \Omega_A(k')\Omega_B(N-k')}$ for regions A and B, respectively. These probabilities, which correspond to the histogram that one could accumulate in a set of infinitely long (perfect sampling) simulations at fixed number of particles, are then

used to infer estimates for Ω_A and Ω_B using Algorithm S1. Figure S2 shows the exact and inferred Ω for the two regions. The inference is exact when $k \leq N_{max}$. However, since we are in a regime where none of the analyzed simulations has more than N_{max} particles, the method has no way to infer the partition function of $k > N_{max}$.

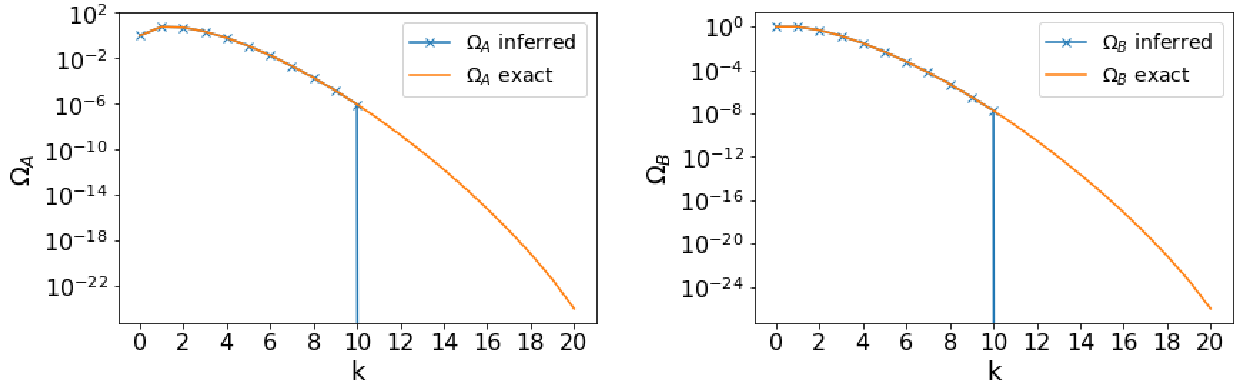

(a) (b)

Figure S2: Inferred and exact values of the partition functions (a) Ω_A of region A and (b) Ω_B of region B, with infinite sampling and number of particles $N_{max} = 10$ lower than the number of lattice sites, $S = 40$. Whereas for $k \leq N_{max}$ the inference is exact, the inferred $\Omega_{A/B}(k) = 0 \; \forall k > N_{max}$.

We then use the inferred partition functions to compute the distribution of particles in region A at fixed concentration of particles in region B. This requires to first estimate the chemical potential μ associated to the concentration in region B using Algorithm S2, and then computing the probability to observe k particles in region A using the grand-canonical weights at that chemical potential. Figure S3a shows the estimated average number of particles in each region at fixed chemical potential. The inferred number of particles agrees very well with the analytical solution for values of μ corresponding to a number of particles smaller than $N_{max} = 10$. However, at a chemical potential low enough to lead to more than 10 particles per region, the inference is incorrect and predicts only 10 particles per region at most. The inferred partition functions are then used to compute the distribution of the number of particles in region A for a list of 5 different concentrations, which correspond to a growing average number of particles in region B (Figure S3, panels b–f). When the

concentration corresponds to an average number of particles in B significantly smaller than $N_{max} = 10$ (panels b–d), the agreement between the inferred and exact solution is virtually perfect. The limitations of the method are clear in cases where the exact distribution would imply a non neglibigle probability to observe more than 10 particles in region A (panel e). Panel f represents the extreme case, where the inferred chemical potential is $-\infty$ and the inferred distribution only allows 10 particles in region A. This test highlights that results can only be reweighted to concentrations that are compatible with the number of particles included in the analyzed simulations.

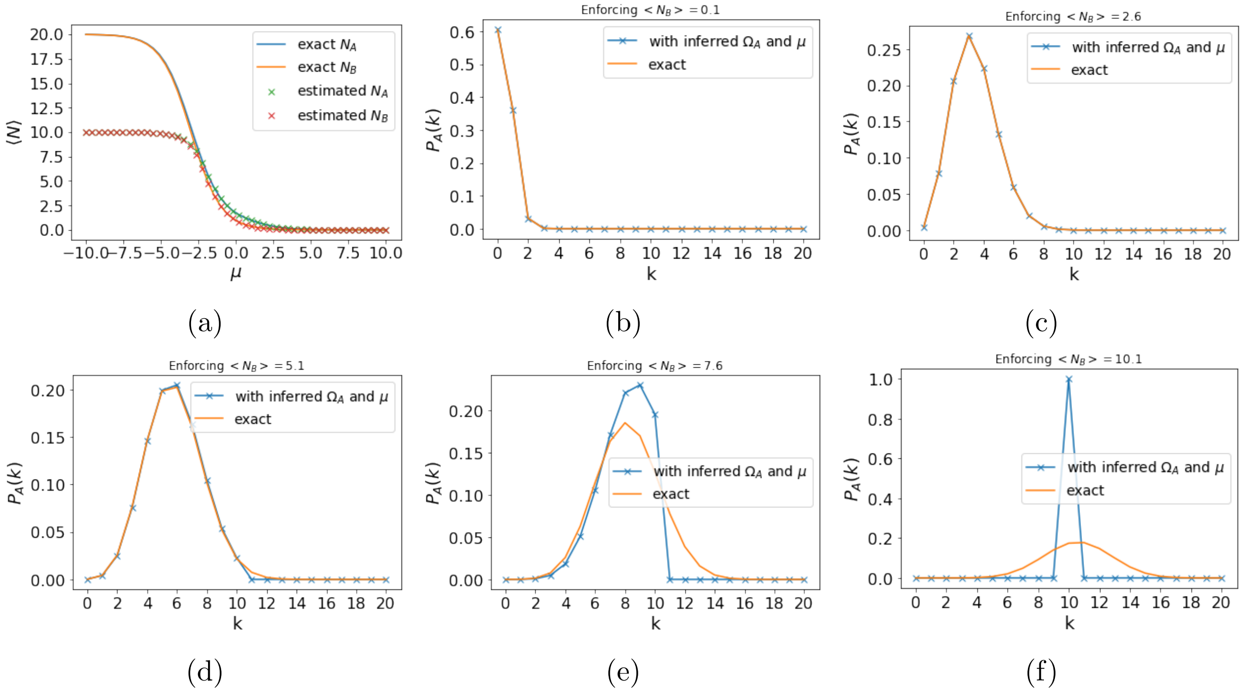

Figure S3: Limitations in the estimates of (a) chemical potential at desired concentrations $\mu(N_B)$ in region B, and (b-f) of the probability distribution of the number of particles in region A at different values of the enforced concentration in B, as tested on the lattice model with a stabilizing site in region A.

Next, we address the issue of obtaining histograms using a finite number of samples. To this aim, instead of assuming to have access to the exact A_k and B_k, we estimate them by drawing $L = 100$ samples from the exact probability distributions. In other words, we consider N_{max} simulations accumulating 100 independent samples. To remove the limitation associated to the finite value of N_{max} discussed above, we here consider $N_{max} = 20$. We first

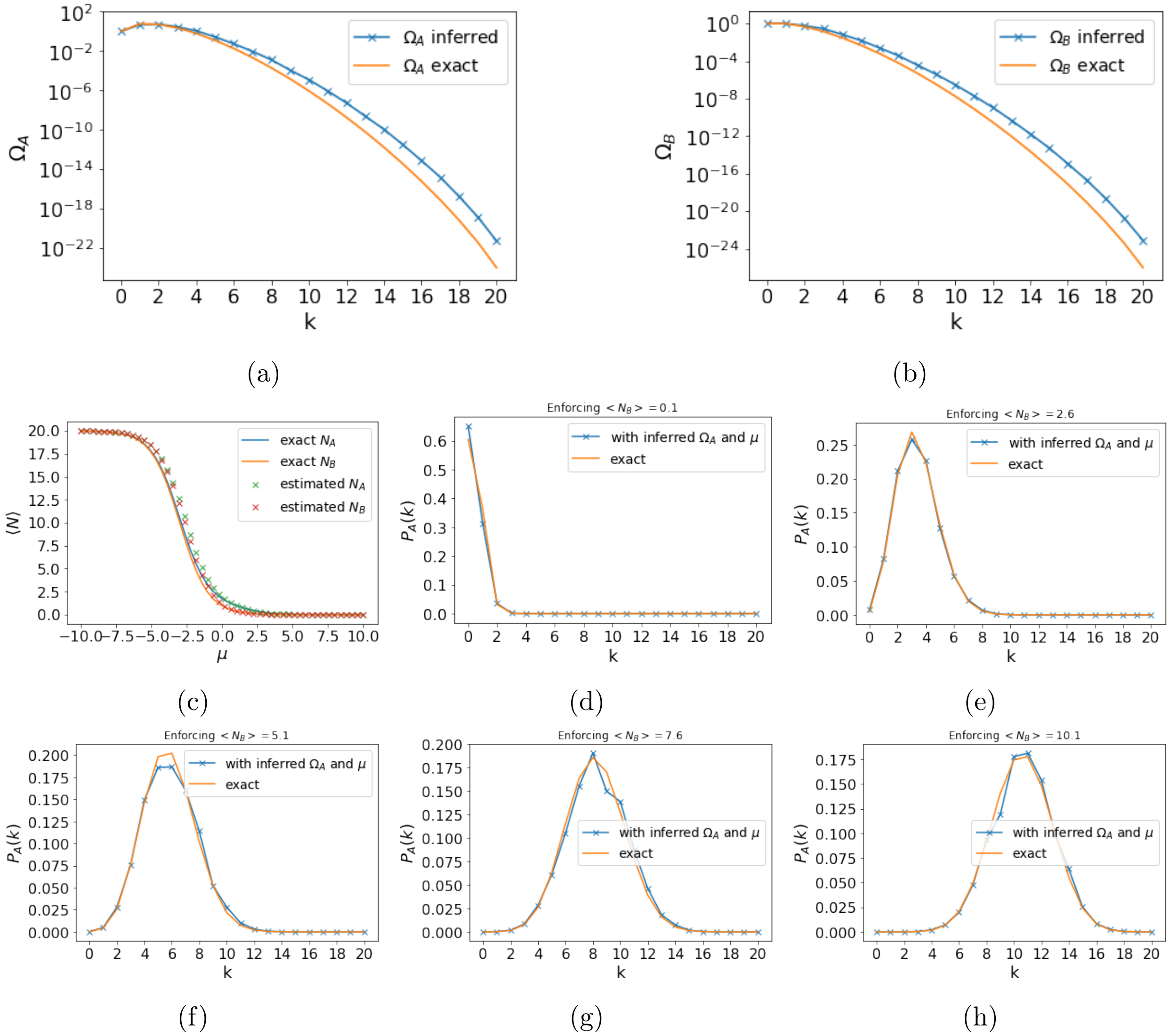

Figure S4: Finite sampling effects in the estimations of (a) $\Omega_A(k)$, (b) $\Omega_B(k)$, (c) the relation between chemical potential μ and number of particles in region A and B, and of (d-h) the probability distribution $P_A(k)$ of the number of particles in region A, as tested on the lattice model with a stabilizing site in region A.

show the inferred partition functions (Figure S4a and b). Since the number of simulated particles is sufficient to cover all cases, no discontinuity is observed. However, the inferred Ω does not match anymore the exact reference. To have an idea of how much this error on the inference of Ω would affect the final result, we use the inferred Ω to estimate the dependence of the average number of particles on μ (Figure S4c). The impact is minimal now, since a sufficient number of particles have been included. The resulting estimates for the population of region A at different particle concentrations are only slightly affected (panels d–h). Clearly, this effect would be larger if the number of samples per simulation was chosen to be smaller.

S4 Parametrization of 1M7

The partial charges of 1-methyl-7-nitroisatoic anhydride (1M7) computed through RESP as described in the main text, are reported in Table S1

Table S1: Charges of atoms in the 1M7 topology as obtained via Antechamber using the RESP method.

Atom	Charge (e)
C1	0.013677
H1	0.165938
C2	-0.301276
H2	0.214747
C3	0.213481
N2	0.680265
O4	-0.418660
O5	-0.418660
C4	-0.403042
H3	0.221441
C5	0.431676
N1	-0.308580
C8	0.913611
O2	-0.562770
C9	-0.336386
H4	0.158142
H5	0.158142
H6	0.158142
C6	-0.413908
C7	0.925472
O3	-0.538891
O1	-0.552561

S5 Initial conformations

Examples of the initial conformation for simulations with $N = 5$ and $N = 16$ are reported in Fig. S5.

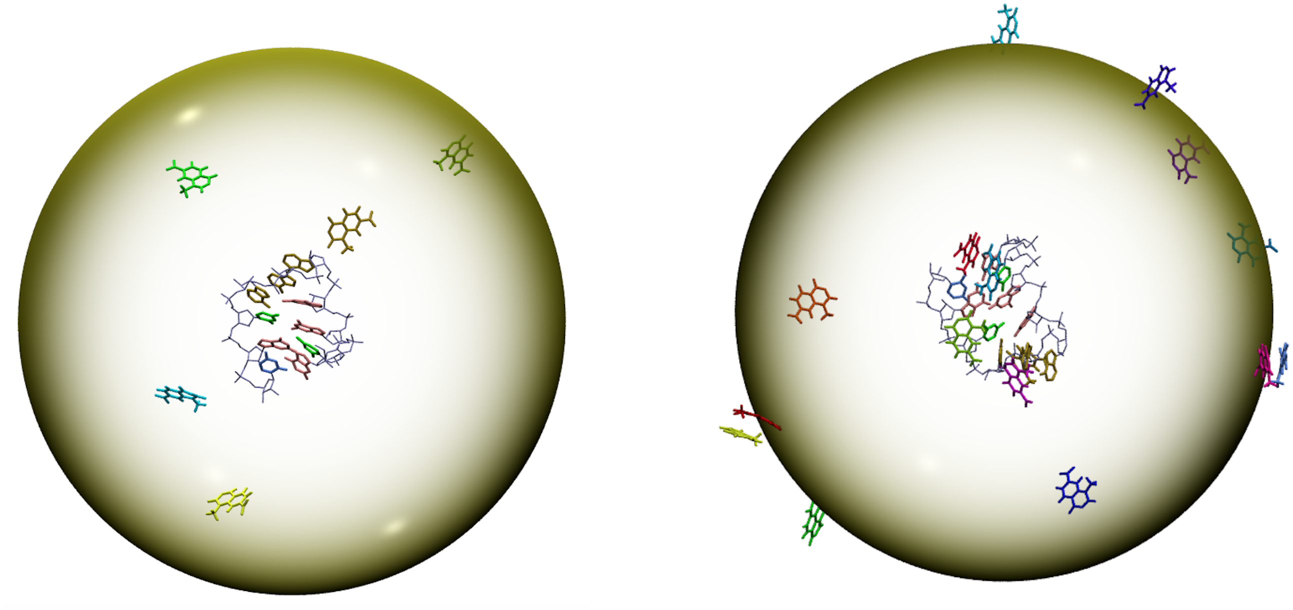

(a) (b)

Figure S5: Two examples of initial conformation for the simulations of the tetraloop with (a) $N = 5$ and (b) $N = 16$ probes. The yellow sphere represents the surface around the tetraloop where probes are initially placed. The RNA tetraloop is at center of the sphere and is shown in thin sticks representation, with nucleobases highlighted as thicker sticks and with different colors. Different 1M7 molecules are displayed with different colors.

S6 Conformational dynamics as a function of reagent concentration

We here report an analysis of the conformational dynamics at varying reagent concentration. For each value of reagent concentration, we subsampled 1000 frames from the total trajectory, with grand canonical ensemble weights $w(N_A)$ that depend on the number of reagent copies in each frame. Frames are sampled with the constraint that no nucleotide in the RNA molecule is under probing (i.e. no reagent copy is in the binding region of any nucleotide). Notice that reagents in the vicinity of RNA might alter the RNA ensembles also without a direct contact with the hydroxyl groups. We computed the eRMSD deviation of all the sampled frames with respect to a common reference structure, at each value of reagent concentration. Additionally, we explored the dynamic extended secondary structures corresponding to each concentration. We observed a slight increase in the median and variance of the eRMSD with respect to the reference at concentrations $C \geq 5\,\mathrm{mM}$, corresponding to a decrease in the ensemble frequency of the G74-A77 trans-Sugar/Hoogsten edge base-pair, compensated by the emergence of C78-G79 ($C \geq 5\,\mathrm{mM}$) and C72-G73 ($C \geq 8\,\mathrm{mM}$) upward stacking. These observations are consistent with the fact that chemical probes can affect the tetraloop structure. More significant changes are observed when considered the ensembles constructed by assuming one or more reagents directly bound to RNA in a position that would allow acylation to occur, as discussed in the main text.

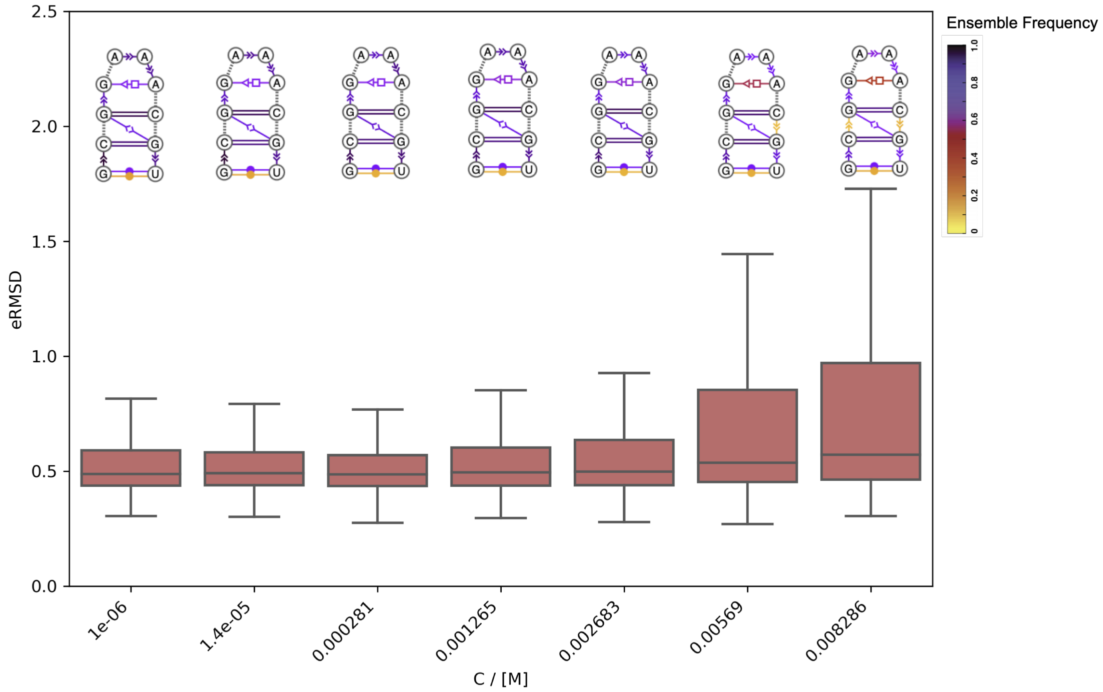

Figure S6: eRMSD deviation from a common reference structure and dynamic secondary structures at varying reagent concentration show minor changes in probability of annotated interactions (base-pairs and base-stacks) within grand-canonical reweighted ensembles.

S7 Conformational dynamics under probing

Here we report the extended dynamic secondary structures and all the computed eRMSD distances for all the probing settings, including those involving nucleotides that do not show significant cooperative behavior.

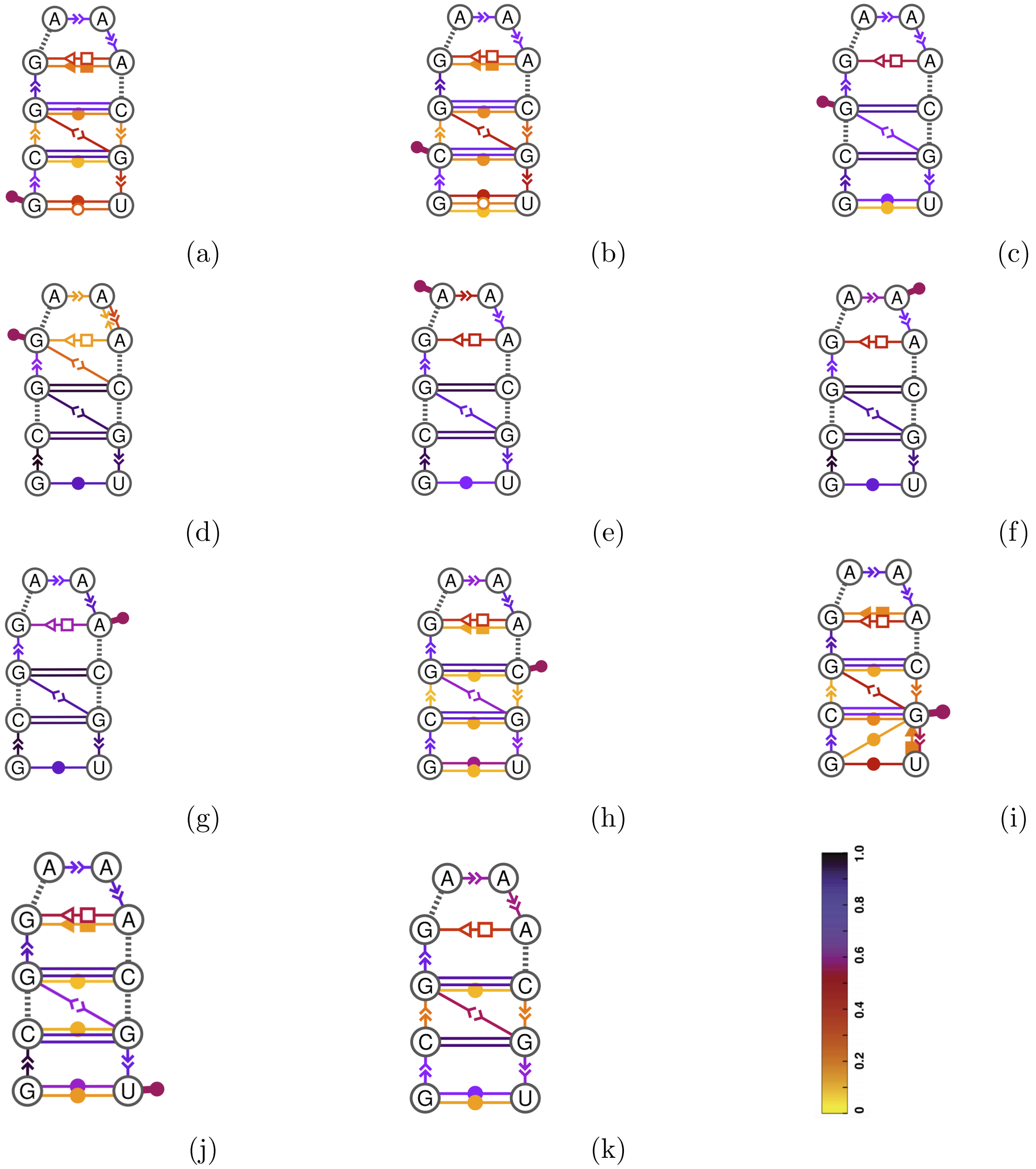

Figure S7: Dynamic secondary structures showing the probability of annotated interactions within grand-canonical reweighted ensembles constrained by individual binding of each nucleotide. Binding is represented through a sketch of the reagent (in red). The bound nucleotides are (a) G71 (b) C72, (c) G73, (d) G74, (e) A75, (f) A76, (g) A77, (h) C78, (i) G79, (j) U80. (k) The same analysis with no constraint on binding. Base pairings are displayed using the Leontis-Westhof. The colormap indicates the population of each of the annotated interactions.

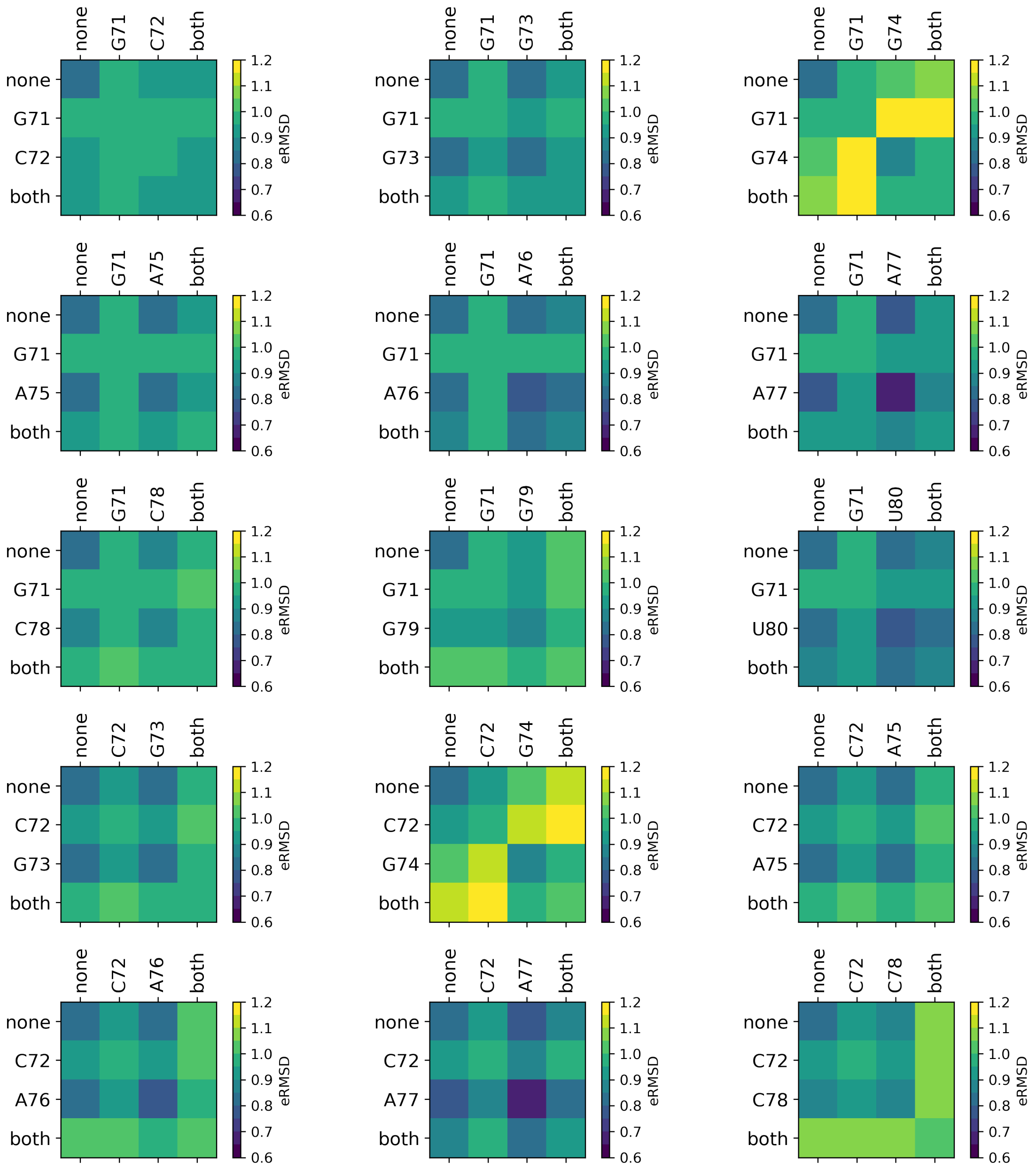

Figure S8: Matrices of root mean square eRMSD deviations between the trajectories sampled with constraints of individual and simultaneus binding for all pairs of nucleotides (G71 to C72).

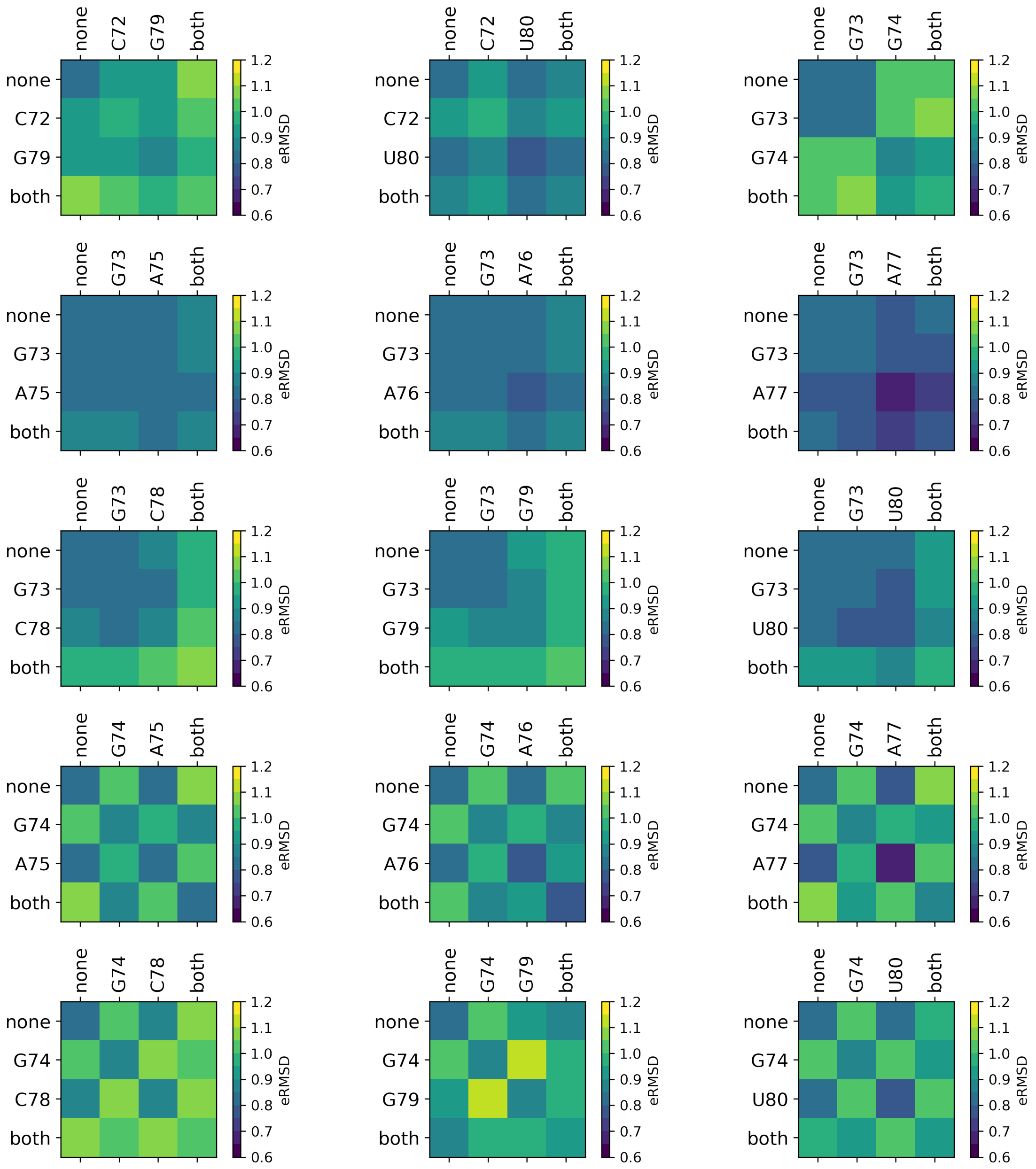

Figure S9: Matrices of root mean square eRMSD deviations between the trajectories sampled with constraints of individual and simultaneus binding for all pairs of nucleotides (C72 to G74).

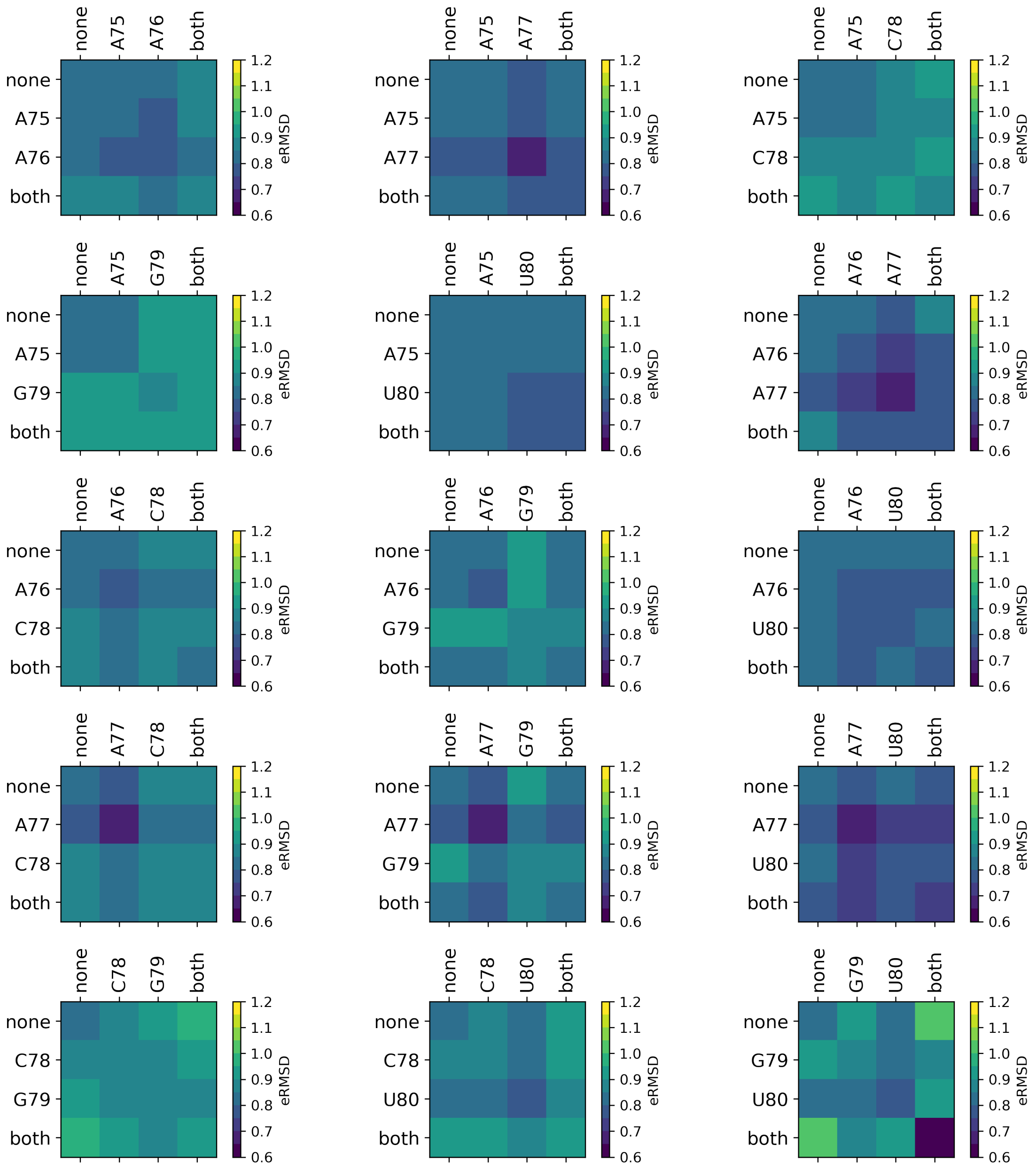

Figure S10: Matrices of root mean square eRMSD deviations between the trajectories sampled with constraints of individual and simultaneus binding for all pairs of nucleotides (A75 to G79).

S8 Effect of grand-canonical reweighting

Here we compare results obtained estimating the reactivities from simulations at constant number of particles with reactivities obtained from the reweighting of the concatenated trajectories according to the grand-canonical ensemble. Figure S8 shows that results are qualitatively comparable in a range where the number of reagent copies has been chosen consistently with the enforced reagent concentration. However, an important advantage of the grand-canonical reweighting procedure is that the behavior as a function of concentration is much smoother than the behavior as a function of the number of reagent copies. This is expected, since individual trajectories are subject to statistical errors. The possibility to compute weighted averages from a single set of trajectories thus makes the estimation of concentration-dependent quantities more statistically robust.

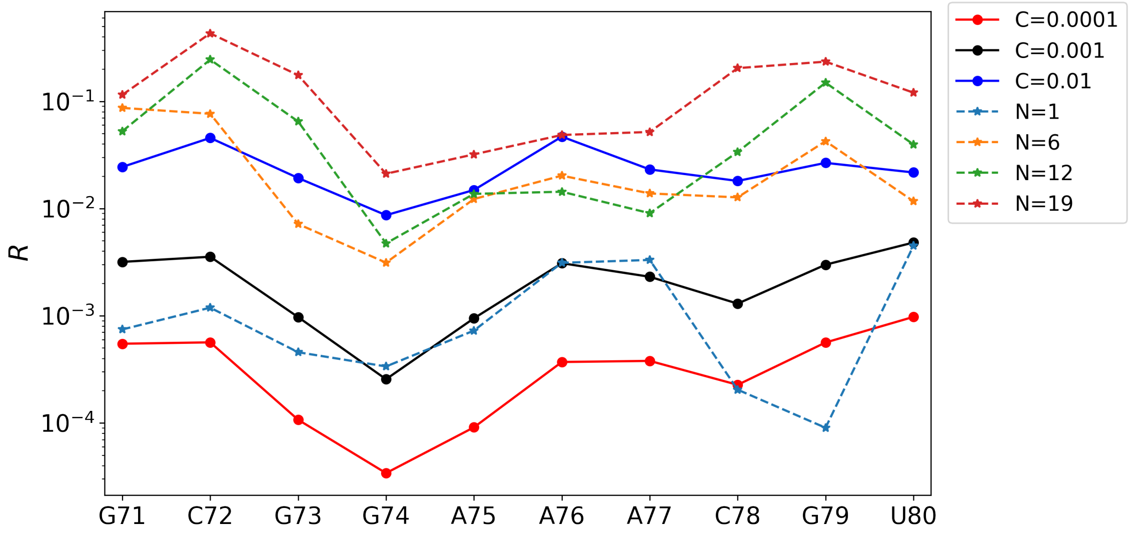

Figure S11: Reactivity profiles from simulations at fixed number of copies $N = 1$, 6, 12, 19 and at fixed concentrations $C = 10^{-1}$ mM, 1 mM, 10 mM as obtained through grand-canonical reweighting.

S9 Effect of helix length

As a control on the possible bias introduced by the specific choice of the length of the simulated helix, we generated a trajectory with an extended portion of the SAM-I riboswitch gcgGAAAcgu tetraloop, namely ranging from C69 to G82 (two additional base-pairs C69-G82 and A70-U81), fixing the number of reagent copies to the representative value $N = 6$. We thus compared the reactivity profile computed from this control simulation with those reported in our main study. As shown in Fig. S12, the reactivity profile of the longer helix was consistent with the fluctuactions associated with the varying number of reagent copies when simulating the shorter helix, as well as the average reactivity of nucleotides in the loop.

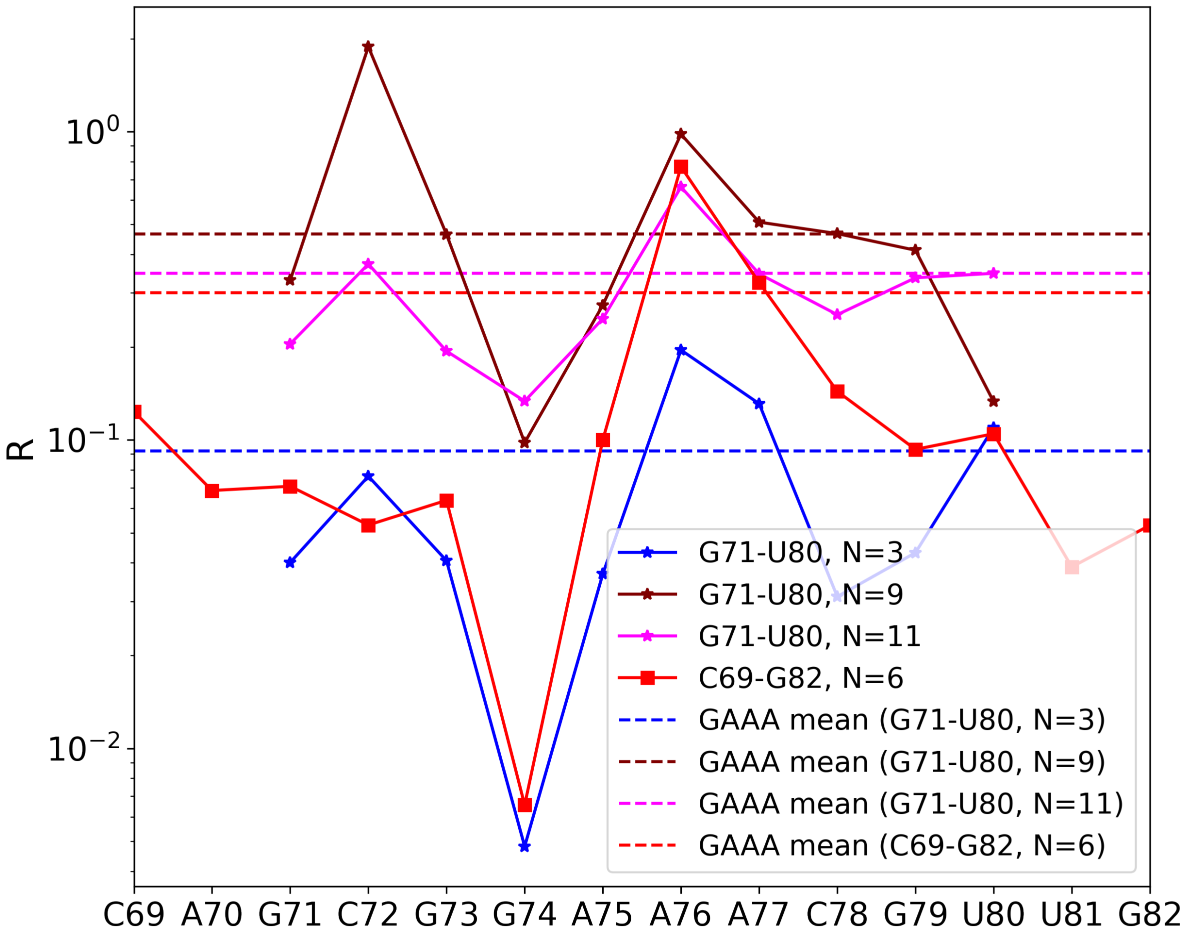

Figure S12: Comparison of reactivity profiles at fixed number of reagent copies $N = 6$ between a longer C69-G82 helix and the shorter G71-U80 helix used in the main study.

S10 Effect of ionic conditions

As a control of the possible effects of different ionic conditions on the cooperative binding dynamics of RNA with 1M7, we generated a trajectory at number of reagent copies fixed to a representative value of $N = 6$, in which the simulation environment contained additional 26 Cl^-/Na^+ ion pairs, corresponding to a nominal concentration of $0.10\,M$, in such a way that the total charge of the system was preserved. As shown in Fig. S13, changes in the reactivity profile obtained from the control simulation were of the order of differences associated with small (±1) variations of the number of reagent copies. This result is expected since SHAPE reagents are neutral, hence their distribution around RNA should not be highly affected by the effective electrostatic screening.

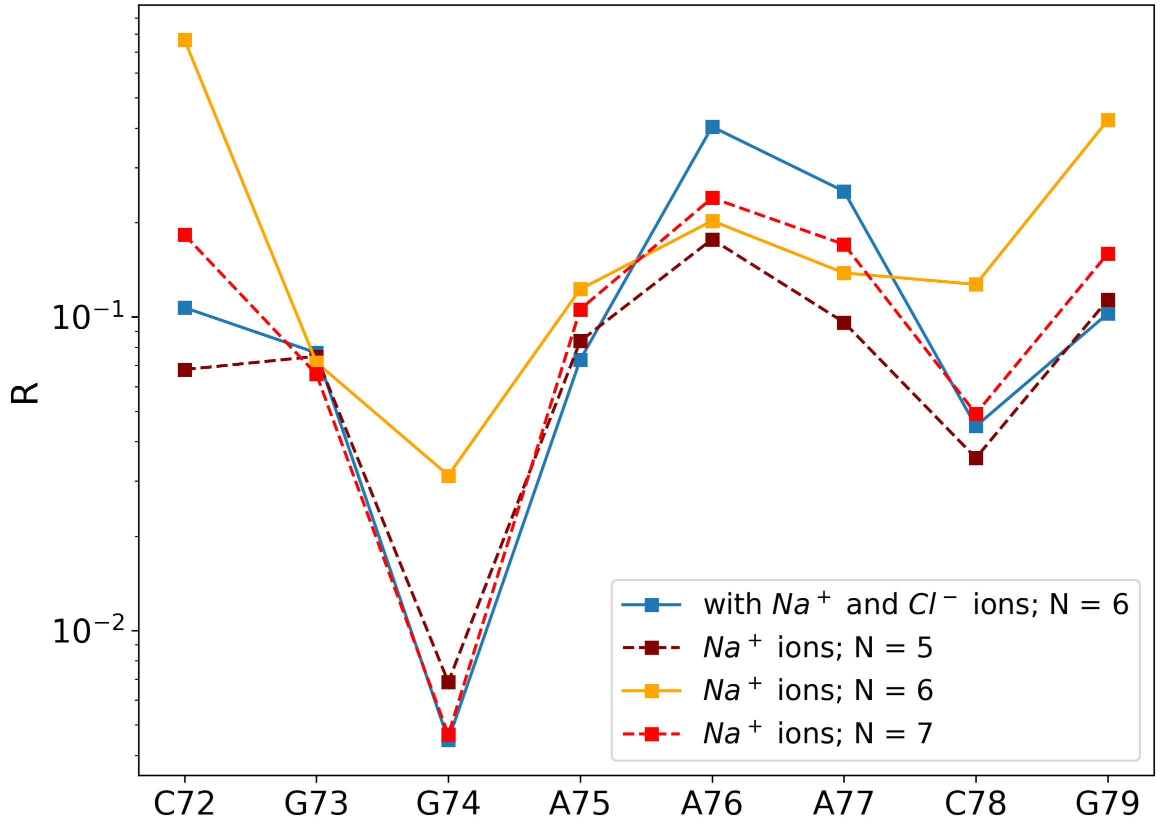

Figure S13: Comparison of reactivity profiles at fixed number of reagent copies $N = 6$ between two different ionic conditions: with only Na^+ ions (blue) and with ions Na^+ and counter-ions Cl^- (orange). As further control, reactivities in presence of only Na^+ ions with $N = 5$ (maroon) and $N = 7$ (red) reagent copies are reported.
